# Bifurcated Polymorphic Transition and Thermochromic Fluorescence of a Molecular Crystal Involving Three-Dimensional Supramolecular Gear Rotation

**DOI:** 10.1021/jacs.3c12454

**Published:** 2024-03-12

**Authors:** Yun-Hsuan Yang, Yu-Shan Chen, Wei-Tsung Chuang, Jye-Shane Yang

**Affiliations:** † Department of Chemistry, National Taiwan University, Taipei 10617, Taiwan; ‡ National Synchrotron Radiation Research Center, Hsinchu 30092, Taiwan

## Abstract

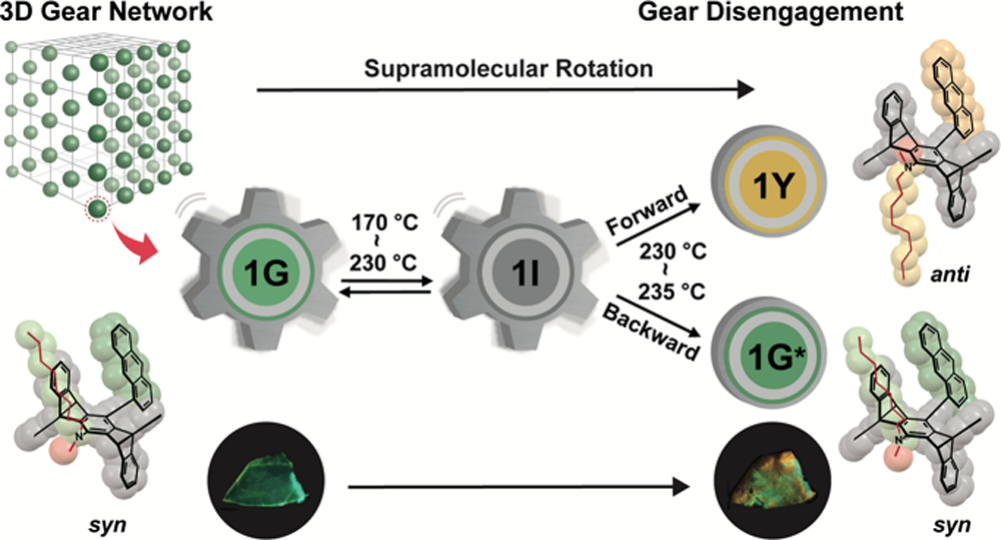

The ability of molecules to move and rearrange in the solid state accounts for the polymorphic transition and stimuli-responsive properties of molecular crystals. However, how the crystal structure determines the molecular motion ability remains poorly understood. Here, we report that a three-dimensional (3D) supramolecular gear network in the green-emissive polymorph **1G** of a dialkylamino-substituted anthracene-pentiptycene π-system (**1**) enables an unusual bifurcated polymorphic transition into a yellow-emissive polymorph (**1Y**) and a new green-emissive polymorph (1G*) via 3D correlated supramolecular rotation. The 90° forward correlated rotation causes the molecular conformation between the octyl and the anthracene units to change from *syn* to *anti*, the ladder-like supramolecular columns to constrict, and the gear network to disengage. This cooperative molecular motion is marked by the gradual formation of an intermediate state (**1I**) across the entire crystal from 170 to 230 °C, which then undergoes bifurcated (forward or backward rotation) and irreversible transitions to form polymorphs **1Y** and 1G* at 230–235 °C. Notably, 1G* is similar to **1G** but lacks gear engagement, preventing its transformation into **1Y**. Nevertheless, **1G** can be restored by grinding **1Y** or 1G* or fuming with dichloromethane (DCM) vapor. This work illustrates the correlation between the crystal structure and solid-state molecular motion behavior and demonstrates how a 3D molecular gear system efficiently transmits thermal energy to drive the polymorphic transition and induce fluorochromism through significant conformational and packing changes.

## Introduction

Stimuli-induced solid-state molecular motion and rearrangement form the basis for optical,^1−10^ mechanical (morphological),^11−29^ electrical,^30,31^ and/or topochemical^32−38^ responses of molecular crystals. A mechanistic understanding of how molecules move within the constraints of crystalline lattices and noncovalent interactions is crucial for the rational design of molecular crystals for use in machinery, sensory devices, and organic electronics. However, phase transitions induced by stimuli in organic crystals often lead to the formation of polycrystalline or amorphous structures,^39−41^ rendering the characterization of molecular motions and transformations exceedingly challenging. Therefore, systems that exhibit single-crystal-to-single-crystal (SCSC) transitions^4,6,10,12,14,24,33,37^ or undergo polymorphic transitions^8,9,23,30,42^ are valuable candidates for gaining insights into molecular mobility in the solid state.

Identifying molecular trajectories for the transformation from the parent to the daughter phase is by no means an easy task, particularly when significant structural changes have occurred.^43−45^ Solid-to-solid molecular phase transitions are generally categorized into two types: nucleation-and-growth (reconstructive) transitions and cooperative (martensitic) transitions.^46^ Systems with a cooperative mechanism typically exhibit rapid and reversible SCSC transitions, involving small degrees of structural changes, such as the rotation of subunits.^46−48^ In contrast, nucleation-and-growth transitions allow for larger amplitudes of molecular reorientation, translation, and/or conformational rearrangement, albeit at lower rates and reversibility.^49−51^ A key distinction between these two mechanisms is the molecular motion being one-by-one (nucleation-and-growth) or layer-by-layer (cooperative).^46^ However, the boundary between them may not always be clear-cut,^52−54^ and the operation of these two mechanisms may not always be mutually exclusive.^9,54^ Furthermore, while phenomena like mechanically coupled motions, such as gear rotation of molecular subunits, have been demonstrated in molecular crystals,^55^ phase transitions driven by mechanically coupled molecular motions have not yet been reported. Additionally, there are examples of sequential or condition-controlled polymorphic transitions among three or more polymorphs.^54,56−58^ However, a bifurcated polymorphic transition, where a polymorph simultaneously splits into two distinct polymorphs, remains unknown.

In this paper, we present a mechanically coupled cooperative molecular motion that drives the bifurcated polymorphic transition of a molecular crystal, resulting in two polymorphs of distinct molecular conformational and crystal packing. Specifically, the green-emissive polymorph (**1G**) of the *N*-methyl-*N*-octylamino-substituted anthracene–pentiptycene π-system **1** can be thermally transformed into a yellow-emissive polymorph (**1Y**) and a new green-emissive polymorph (1G*). The **1G**-to-**1Y** transformation is achieved through a 90° three-dimensional (3D) correlated rotation of the supramolecular dimers and columns. This rotation changes the molecular conformation between the octyl chain and the anthracene group from *syn* to *anti*, causes the ladder-like supramolecular columns to constrict, and alters the crystal system from monoclinic to triclinic. The origin of the 3D correlated supramolecular motion lies in the 3D gear network in **1G**. In this network, the H-shaped pentiptycene unit^59^ and the *N*-methyl-*N*-methylene (CH_3_–N–CH_2_) moiety act as the rigid and rotatable teeth, respectively. We observed a characteristic intermediate state (**1I**), which facilitates bifurcated polymorphic transitions to **1Y** and 1G* through forward and backward correlated rotation, respectively. The disengagement of the gear network in the daughter phases **1Y** and 1G*, highlights the irreversibility of the polymorphic transition. Nevertheless, the restoration of **1G** can be achieved through mechanical grinding or exposure to dichloromethane (DCM) vapor. Our results not only illustrate the correlation between crystal structure and solid-state molecular motion but also demonstrate the potential of utilizing gear rotation in solid-state molecular devices.

## Results and Discussion

**Scheme 1:**
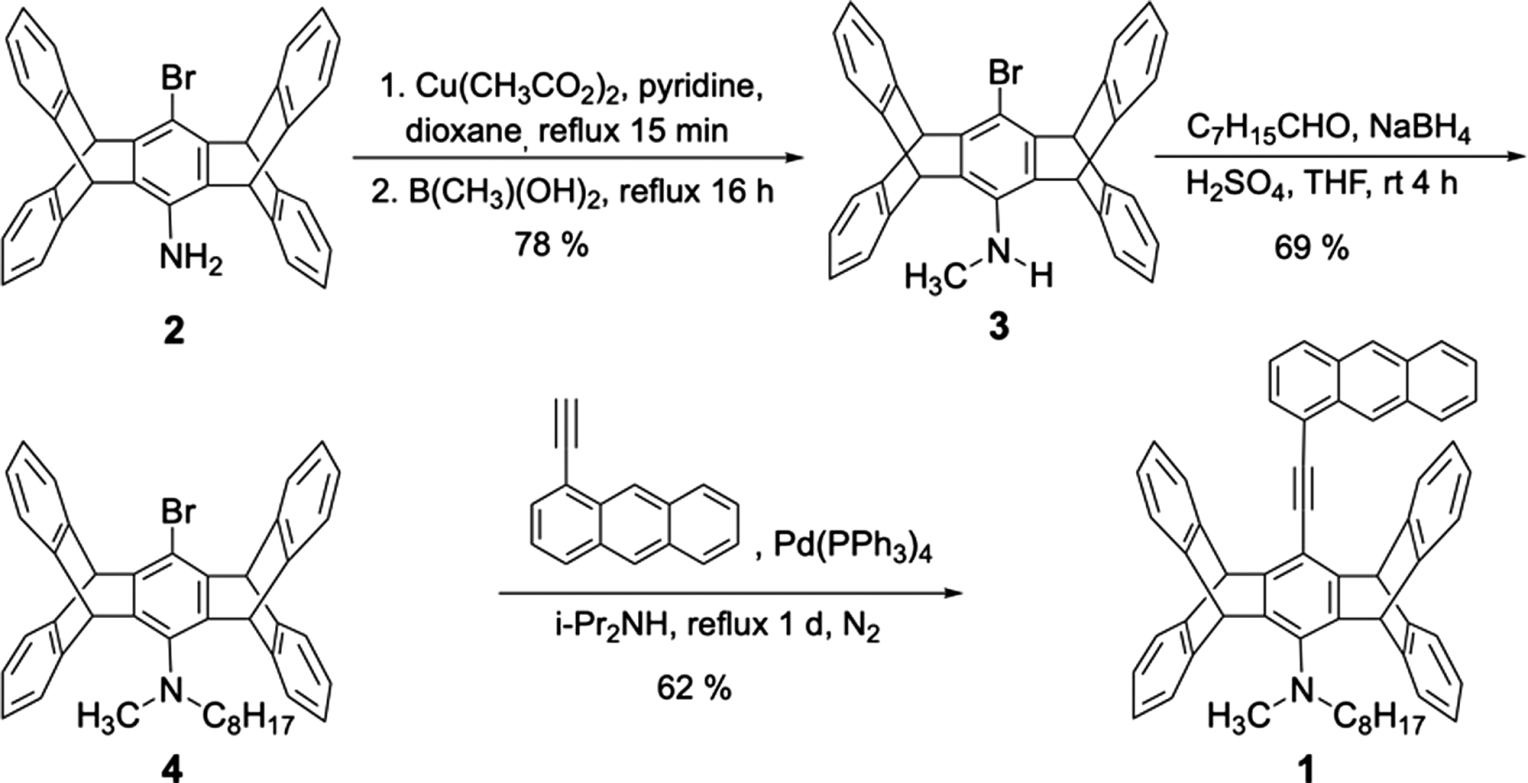
Synthesis of **1**

**Synthesis and Solution Properties.** The anthracene-pentiptycene π-system **1** is investigated by following the intriguing polymorphism and stimuli-responsive fluorescence and mechanical properties of its alkyloxy-substituted counterparts.^60−62^ Since alkyl-pentiptycene C–H···π interactions play a critical role in determining the crystal packing mode of pentiptycene derivatives,^60−64^ we hypothesize that the presence of one additional alkyl group for -NRR′ vs -OR units could modify the crystal structures and hence induce new stimuli-responsive properties. Scheme 1 shows the synthesis of **1** from the known building block **2**(65) via intermediates **3** and **4** though *N*-alkylation and Sonogashira coupling reactions. The details of the synthetic procedures and compound characterization data are available in the Supporting Information. The absorption spectrum of **1** shows little dependence on solvents and displays vibrational bands at 382 and 401 nm (Figure S1). However, its emission spectra exhibit positive solvatofluorochromism with a shift of ∼5500 cm^–1^ on going from hexane (λ_f_ = 437 nm) to acetonitrile (λ_f_ = 574 nm). The fluorescence quantum yield (Φ_f_) falls within the range 0.19–0.50, depending on the nature of the solvents (Table S1).

**Figure 1:**
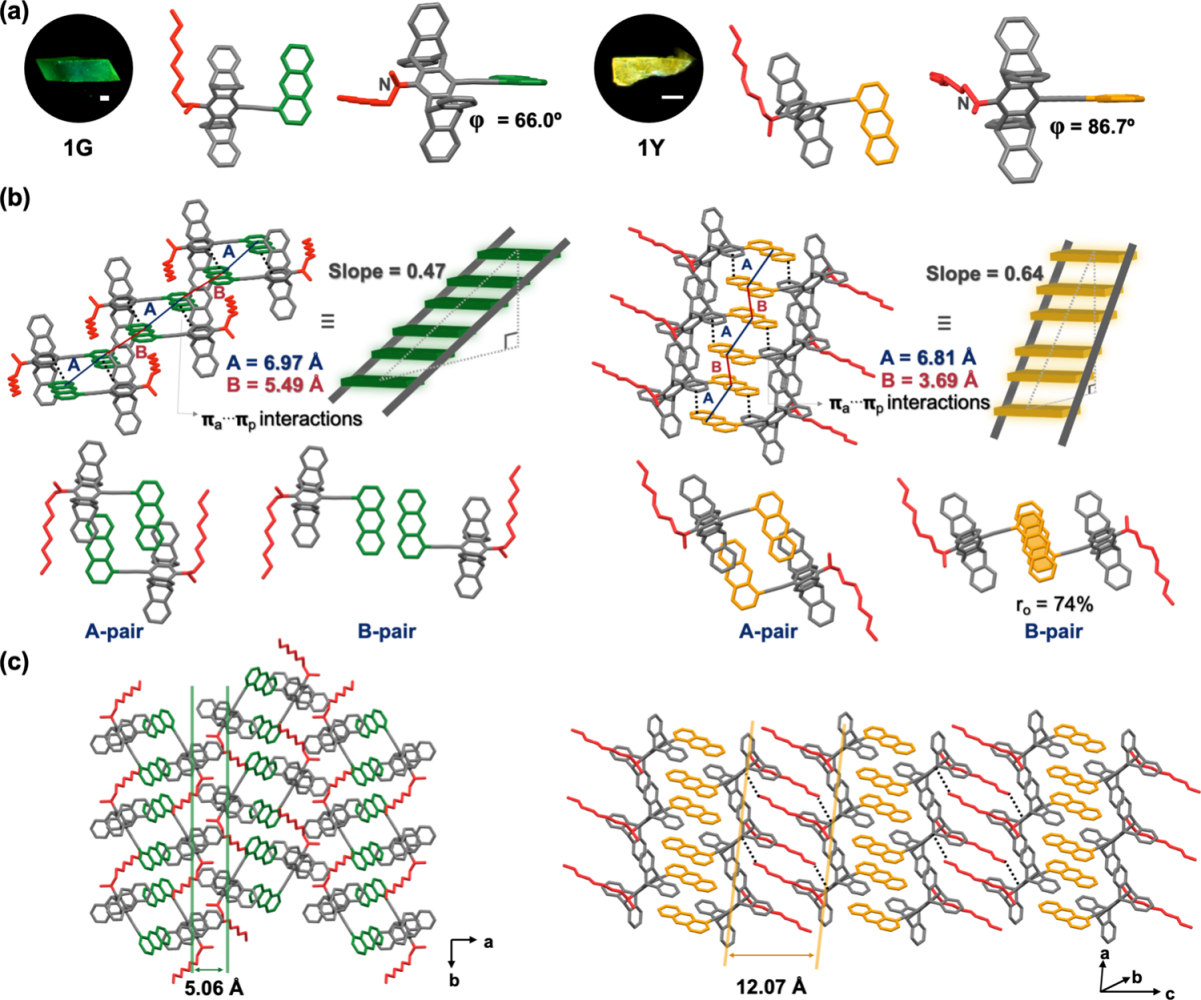
Crystal polymorphism of compound **1** (**1G**, left, and **1Y**, right): (a) fluorescence microscopy images (scale bars: 200 μm) and molecular structures depicting the *syn* and *anti* orientation of the octyl chain relative to the anthracene unit; (b) ladder-like supramolecular column showing the slope and the A- and B-pair component with the π_a_–π_p_ and/or π_a_–π_a_ interactions; (c) a supramolecular sheet formed by the ladder-like supramolecular columns, illustrating distinct intercolumnar distances. The dialkylamino group is highlighted in red to emphasize its location and orientation.

**Crystal Polymorphism.** In CH_2_Cl_2_/MeOH (*v*/*v* = 1/1) mixed solvents, **1** mainly crystallizes in a green-emissive form (**1G**, λ_f_ = 541 nm) with a minor part (<5%) of crystals that emit yellow fluorescence (**1Y**, λ_f_ = 587 nm). Both **1G** (Φ_f_ = 0.77) and **1Y** (Φ_f_ = 0.59) exhibit stronger fluorescence compared to **1** in solutions, representing a new example of crystallization-induced emission enhancement.^40,66^ Single-crystal X-ray diffraction (SCXRD) experiments reveal that **1G** and **1Y** are polymorphs and belong to the space groups of *P*2_**1**_/*c* (monoclinic) and *P*1̅ (triclinic), respectively (Table S2). A significant difference in molecular conformation between **1G** and **1Y** is the orientation of the *N*-octyl chain relative to the anthracene group: the *syn* form in **1G** but the *anti* form in **1Y** (Figure 1a). In addition, the pentiptycene-amino C–N torsion angle (φ) is larger in **1Y** (φ = 86.7°) than that in **1G** (φ = 66.0°). Regarding the packing mode, both **1G** and **1Y** adopt an antiparallel arrangement to form a supramolecular dimer (A-pairs in Figure 1b) with anthracene-pentiptycene π–π interactions (π_a_–π_p_ interactions), which is characterized with a tongue-and-groove-like packing of anthracene in the pentiptycene U-shaped cavity.^64^ The one-dimensional packing of these supramolecular dimers creates a columnar structure resembling a ladder, where the anthracene groups serve as the rungs, and the pentiptycene moieties form the stiles. However, the rungs are not evenly spaced but arranged alternately in an ABAB pattern, and the steps’ slope is steeper in **1Y** (0.64) than in **1G** (0.47). In **1G**, adjacent A-pairs within the same column exhibit octyl-anthracene C–H···π (CH_o_–π_a_) interactions (Figure S2). The anthracene–anthracene distance is 6.97 Å for the A-pairs and 5.49 Å for the B-pairs, indicating negligible anthracene–anthracene π-stacking (π_a_–π_a_) interactions. The supramolecular columns are arranged side by side through *N*-methyl-pentiptycene C–H···π (CH_m_–π_p_) interactions with an intercolumnar spacing of 5.06 Å (Figure 1c). The absence of π_a_–π_a_ interactions in **1G** suggests monomer-like emission. In contrast, the supramolecular columns in **1Y** exhibit π_a_–π_a_ interactions (the B-pairs) with a plane-to-plane distance (*d*_π_) of 3.50 Å, center-to-center distance (*d*_c_) of 3.69 Å, and overlap ratio (*r*_o_) of 74%, in addition to the π_a_–π_p_ interactions in the A-pairs. Notably, the intercolumnar spacing is significantly larger in **1Y** (12.07 Å), and the terminal carbon of the octyl chain interacts with the pentiptycene of the neighboring columns (Figure 1c). The π_a_–π_a_ interactions contribute to the red-shifted fluorescence and the [4 + 4] photodimerization activity^60−63^ of **1Y** relative to **1G**. The photodimerization activity of **1Y** is demonstrated by the presence of approximately 30% of photodimer in the determined crystal structure of **1Y** (Figure S3). The formation of the photodimer resulted from inevitable short-term exposure of the crystals to UV light to allow for the selection of **1Y** out of the batch of mixed **1G** and **1Y**. An independent experiment with long-term (60 min) irradiation of **1Y** led to 85% photodimerization of **1** (Figure S4).

**Figure 2:**
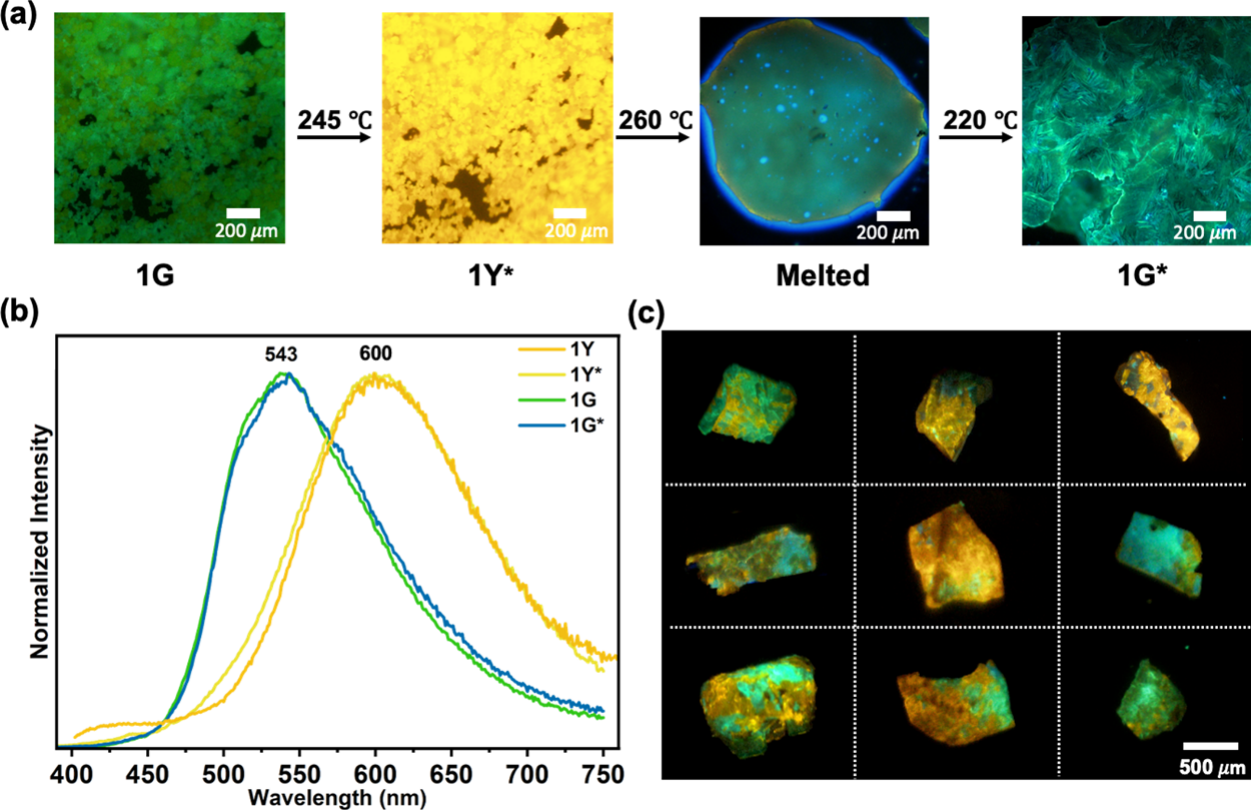
Thermochromic fluorescence of **1G**: (a) fluorescence images of polycrystalline powder; (b) normalized fluorescence spectra of **1Y**, 1Y*, **1G**, and 1G*; (c) fluorescence images of nine single crystals of **1G** after heating to 250 °C, followed by cooling to room temperature.

**Bifurcated Polymorphic Transition of 1G.** Upon heating the polycrystalline powder of **1G** to the temperature range of 220–245 °C, we observed a fluorescence color change from green to yellow (denoted as 1Y*, Figure 2a). The resulting 1Y* melts at ∼260 °C, reverting the fluorescence to green, which persists upon cooling and solidification at ∼220 °C (denoted as 1G*). The fluorescence color of 1Y* and 1G* remains unchanged upon cooling to ambient temperature, indicating an irreversible phase transition. The fluorescence spectra of 1Y* and 1G* resemble those of **1Y** and **1G**, respectively (Figure 2b). Unlike the nearly complete green-to-yellow fluorescence color change mentioned above, heating larger **1G** crystals, ranging from millimeter to submillimeter size, to 250 °C results in the coexistence of both green- and yellow-emissive regions, with the relative fraction varying from one crystal to another (Figure 2c). It is crucial to note that repeated heating and cooling cycles between room temperature and 250 °C for the same crystal do not alter the fluorescence image, confirming that once a phase transition has occurred thermal treatment before melting can no longer alter the resulting phases.

To our delight, both the yellow- and green-emissive regions of the heated single crystals exhibited excellent crystallinity, enabling individual SCXRD characterization. To obtain a pure fragment of either emissive phase for SCXRD analysis, we selected crystals dominated by one of the two phases by removing the minor portion of the other phase. The yellow-emissive region displayed the same space group and density as **1Y** with similar unit cell parameters, whereas the green-emissive region shared the same space group as **1G** but exhibited different unit cell parameters and density (Table 1, see Table S2 for the full data). Combined with powder X-ray diffraction (PXRD) analysis (Figure S5), we conclude that the identities of the yellow- and green-emissive regions in Figure 2c correspond to 1Y* and 1G*, respectively. In addition, **1Y** and 1Y* belong to the same polymorph (Figure S6), and the slight variance in their unit-cell parameters can be attributed to the contamination of photodimer in the **1Y** crystal (vide supra), but not in the 1Y* crystal. In contrast, **1G** and 1G* represent different polymorphs, even though their molecular conformations and crystal packing modes are very similar (Figure S7). These results not only confirm the occurrence of the **1G**-to-**1Y** polymorphic transition but also reveal an unusual bifurcated crystal-to-crystal polymorphic transition, leading to the simultaneous formation of both **1Y** and 1G*. Given the topochemical activity of **1Y** but not **1G**, the **1G**-to-**1Y** phase transition provides a new example of stimuli-induced topochemical responses in molecular crystals.^32−38^

**Table 1:** Space Group and Unit-Cell Parameters of **1G**, 1G*, **1Y**, and 1Y*

crystal	**1G**	1G*	**1Y**	1Y*
space group	*P*2_1_/*c*	*P*2_1_/*c*	*P*1̅	*P*1̅
*a* (Å)	15.4624 (13)	11.7571 (2)	9.2775 (4)	9.1337 (3)
*b* (Å)	11.8288 (12)	12.5138 (3)	11.7412 (5)	11.6033 (4)
*c* (Å)	23.834 (3)	30.0568 (6)	19.9994 (8)	19.8199 (6)
α (degrees)	90	90	80.7852 (19)	81.2028 (12)
β (degrees)	105.495 (10)	106.3240 (9)	82.984 (2)	84.0601 (13)
γ (degrees)	90	90	85.756 (2)	86.5301 (12)
*V* (Å^3^)	4200.8 (8)	4243.87 (15)	2131.00 (16)	2062.54 (12)
*Z* value	4	4	2	2
*D*_cal_ (Mg/m^–3^)	1.221	1.208	1.203	1.203

**Figure 3:**
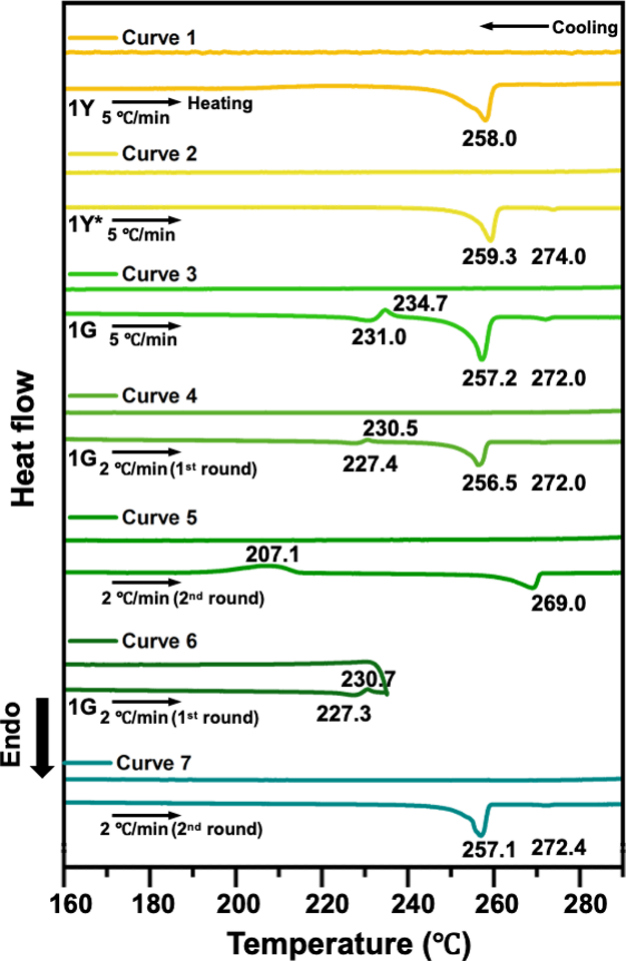
DSC scan of **1Y** at 5 °C/min (curve 1), 1Y* at 5 °C/min (curve 2), **1G** at 5 °C/min (curve 3), **1G** at 2 °C/min: first cycle (curve 4) and second cycle (curve 5), and **1G** at 2 °C/min with heating terminated at 235 °C (curve 6), followed by the second-round heating and cooling (curve 7). Peak temperatures (°C) are indicated near the respective peaks.

The irreversible bifurcated polymorphic transition of **1G** is further confirmed by differential scanning calorimetry (DSC) studies on polycrystalline powders. As shown in Figure 3, both the as-grown **1Y** (curve 1) and the thermally generated 1Y* samples (curve 2) exhibit an endothermic peak near 258 °C, corresponding to their melting points and confirming their common identity. From now on, all the following discussion on 1Y* and **1Y** will be simply referred to as **1Y** only. When **1G** is heated at a rate of 5 °C/min (curve 3), a consecutive endothermic (231 °C) and exothermic (235 °C) process occurs, corresponding to the phase transitions. The subsequent endotherms at 257 and 272 °C can be attributed to the melting of **1Y** (major) and 1G* (minor), respectively. The formation of 1G* is significantly suppressed when the scan rate is reduced to 2 °C/min (curve 4), as evidenced by the weak melting peak of 1G*. This suggests that at the transition temperature **1Y** is thermodynamically more stable than 1G*. Given the lower melting point of **1Y** (257–258 °C) compared to 1G* (270–272 °C), indicating weaker intermolecular interactions (i.e., enthalpy effect), the higher stability of **1Y** vs 1G* at the transition temperature is attributed to an entropy effect. Upon cooling from the melt (curves 1–4), no phase transition was detected in all cases, indicating the formation of an amorphous state. The second-round heating of curve 4 reveals a recrystallization process in the range 186–212 °C, forming only 1G* (curve 5). The thermal irreversibility from **1Y** and 1G* to **1G** is confirmed by terminating the heating scan at 235 °C (curve 6) and subsequently conducting a second-round heating and cooling (curve 7). During this process, no phase transition peaks are observed; only the melting peaks of **1Y** and 1G* are evident. This absence of transition peaks solidly demonstrates that the transformation from **1G** to **1Y** and 1G* is irreversible. In contrast, when heating was halted at 225 °C, the second- and third-round heating and cooling curves mirror those of curves 4 and 5, respectively (Figure S8). This explicitly demonstrates that below the phase transition temperature (230–235 °C), the treatment of **1G** with heating and cooling does not alter its characteristics. Regarding the effect of scan rate on the peak position of the phase transition, the endothermic peak shifts from 235 to 233, 231, and 227 °C with scan rate of 15 to 10, 5, and 2 °C/min, respectively (Figure S9). The phenomenon of scan rate-dependent phase transition temperatures indicates an enantiotropic system.^67^ Therefore, the **1G**-to-**1Y** transformation is a new example of kinetically irreversible enantiotropic transition.^2,9,44,50^ The fact that **1G** (1.221 mg/m^3^) is more stable than **1Y** (1.203 mg/m^3^) at ambient temperature is consistent with the Kitaigorodskii’s density rule: a denser crystal is generally more stable.^68^ Nevertheless, the comparable Δ*H* change in the endothermic and exothermic processes (e.g., 5.2 vs −5.8 J/g at 5 °C/min) of the phase transition indicates that the difference in energy between **1G** and **1Y** is relatively small.

Upon confirmation of the occurrence of irreversible bifurcated polymorphic transitions from **1G** to **1Y** and 1G*, several pertinent questions have emerged. First, the substantial disparities in molecular conformation (*syn*- vs *anti*-form), supramolecular columnar packing (extended vs constricted ladders with small vs large intercolumnar spacing), and crystal symmetry (monoclinic vs triclinic) between **1G** and **1Y** (Figure 1) raise the imperative query: How do the molecules execute such significant molecular motions while upholding high crystallinity and integrity? It is particularly notable that **1G** exhibits a lack of substantial void space (<1% of crystal volume, Figure S10), making any independent molecular motion (i.e., by the nucleation-and-growth mechanism) highly challenging.^69,70^ Second, while the large structural disparities between **1G** and **1Y** might account for the irreversibility of the **1G**-to-**1Y** polymorphic transition, it is a puzzle: Why is the **1G**-to-1G* transition also irreversible, considering their shared *syn* conformation and crystal packing mode? Third, in light of the striking similarity in crystal structures between **1G** and 1G*, why do they behave so differently (e.g., the absence of the 1G*-to-**1Y** transformation) prompts further inquiry. Lastly, the overarching question remains: What is the mechanistic origin of the unusual bifurcated polymorphic transitions of **1G**?

**Figure 4:**
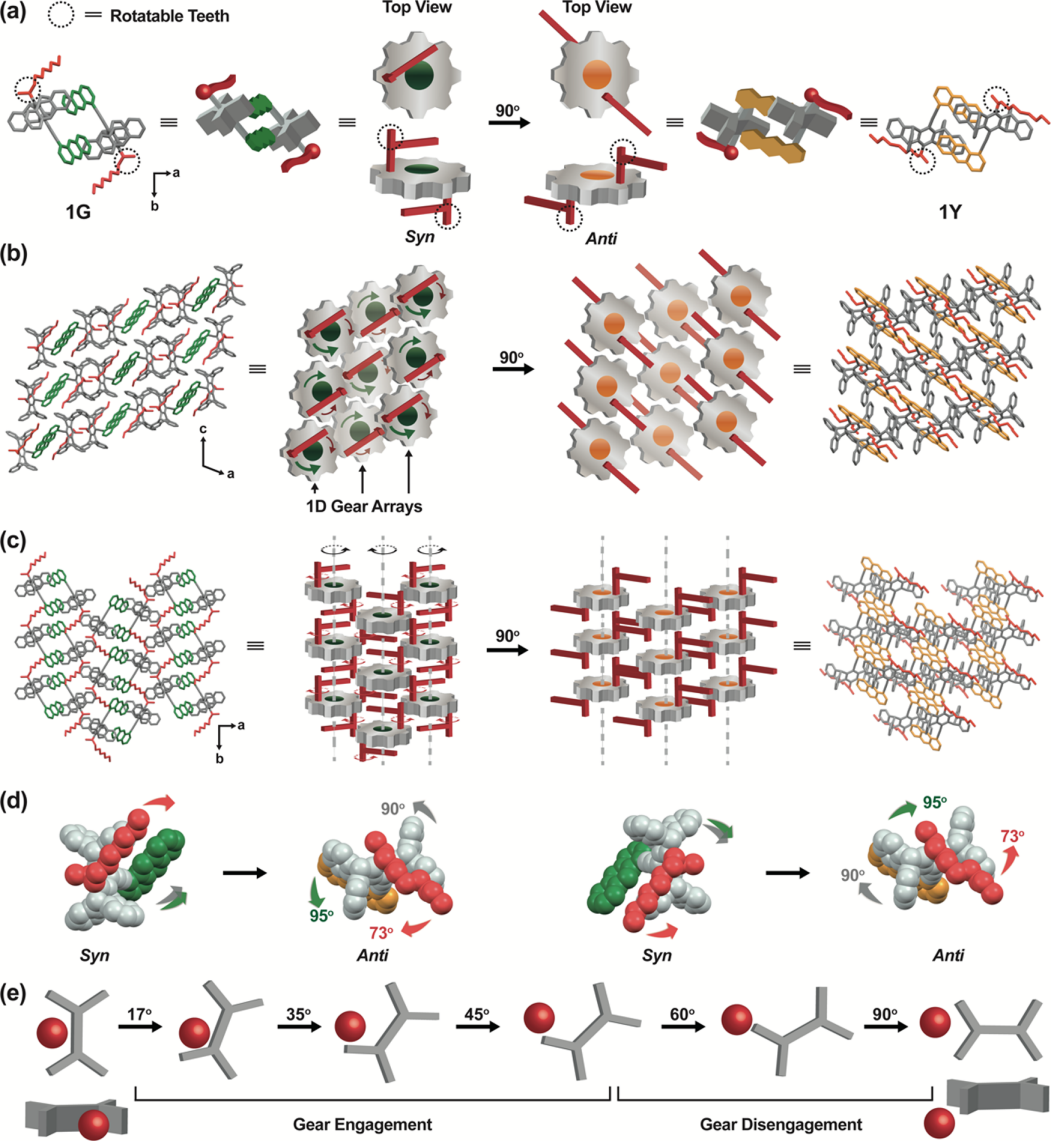
Schematic illustration of the 3D gear model for the **1G**-to-**1Y** transformation: (a) gear-like supramolecular dimer containing both rigid (gray) and rotatable (red) teeth in **1G** (*syn*) and **1Y** (*anti*). (b) 1D gear arrays through the meshing of rigid teeth on the (010) plane in **1G**, where each gear represents the top view of a supramolecular column. The green curved arrows denote the direction of gear rotation, and the red curved arrows denote the direction of the C–N bond rotation. (c) Gear network formed by interlocking of the rotatable teeth with adjacent 1D gear arrays in **1G**. (d) *Syn*-to-*anti* conformational switching along with the counterclockwise (left) and clockwise (right) gear rotation, with the degree of rotation for each component shown. (e) Gear engagement and disengagement of the red tooth in the pentiptycene U-shaped cavity.

**The 3D Gear Model.** To answer these questions, we first conducted a detailed analysis of the crystal structures of the mother (**1G**) and daughter (**1Y**) phases. Our investigations revealed that the crystal structure of **1G** resembles a 3D gear network, where each A-pair supramolecular dimer (Figure 1b) in **1G** functions as a gear. These gears consist of rigid teeth from the pentiptycene blades on the peripheral face and two rotatable teeth from the *N*-methyl-*N*-methylene groups on the face, as depicted in Figure 4a. The rigid teeth mesh with adjacent gears in neighboring columns along the *c* axis, forming one-dimensional (1D) gear arrays (Figure 4b). The 1D gear arrays are integrated through the rotatable teeth of neighboring 1D gear arrays to form a 3D gear network (Figure 4c). As illustrated in Figure 4b,c, a correlated rotation of the gears by 90° about the *b* axis in a disrotatory manner can lead to a transformation in crystal symmetry from monoclinic (**1G**) to triclinic (**1Y**). Note that the rotation direction of the rotatable teeth (the rotating axis is the pentiptycene-amino C–N bond) goes with the meshed gears. Therefore, the rotation direction of the gear and its C–N bonds is opposite, which accounts for the *syn*-to-*anti* conformational transformation. As illustrated in Figure 4d, when the pentiptycene units rotate by 90° counterclockwise, the anthracene group undergoes an additional 5° rotation (i.e., 95° counterclockwise) to facilitate the π_a_–π_a_ interactions, referring to the anthracene-anthracene interactions in the B-pairs of **1Y**. Simultaneously, the C–N bond rotates clockwise by 73°. Consequently, the dihedral angle between the *N*-octyl chain and the anthracene plane undergoes a net change of 168° (i.e., 73° + 95°), leading to the transformation from the *syn* to the *anti* form. Note that Figure 4d also shows another set of gears, where each of the components rotates in the opposite direction but to the same degree. Although a perfect gear rotation would entail both the gear and the C–N bond (the red tooth) rotating by 90° in the opposite direction, the C–N bond experiences a rotation of only 73°. This falls short by 17° due to imperfect meshing, where the tooth pitch (width of the pentiptycene U-shaped cavity) is larger than the tooth thickness (the CH_3_–N–CH_2_ moiety). As schematically depicted in Figure 4e, the geared C–N bond rotation aligns with the gear rotation after the latter have rotated by 17°. The trajectory of this rotation predicts disengagement of the gear network, indicating that the red tooth will fall out of the tooth pitch at a rotation angle of 45°–60°. In summary, the **1G**-to-**1Y** transformation can be successfully explained by a 90° 3D correlated rotation of supramolecular dimers, referred to as the 3D gear model thereafter.

**Figure 5:**
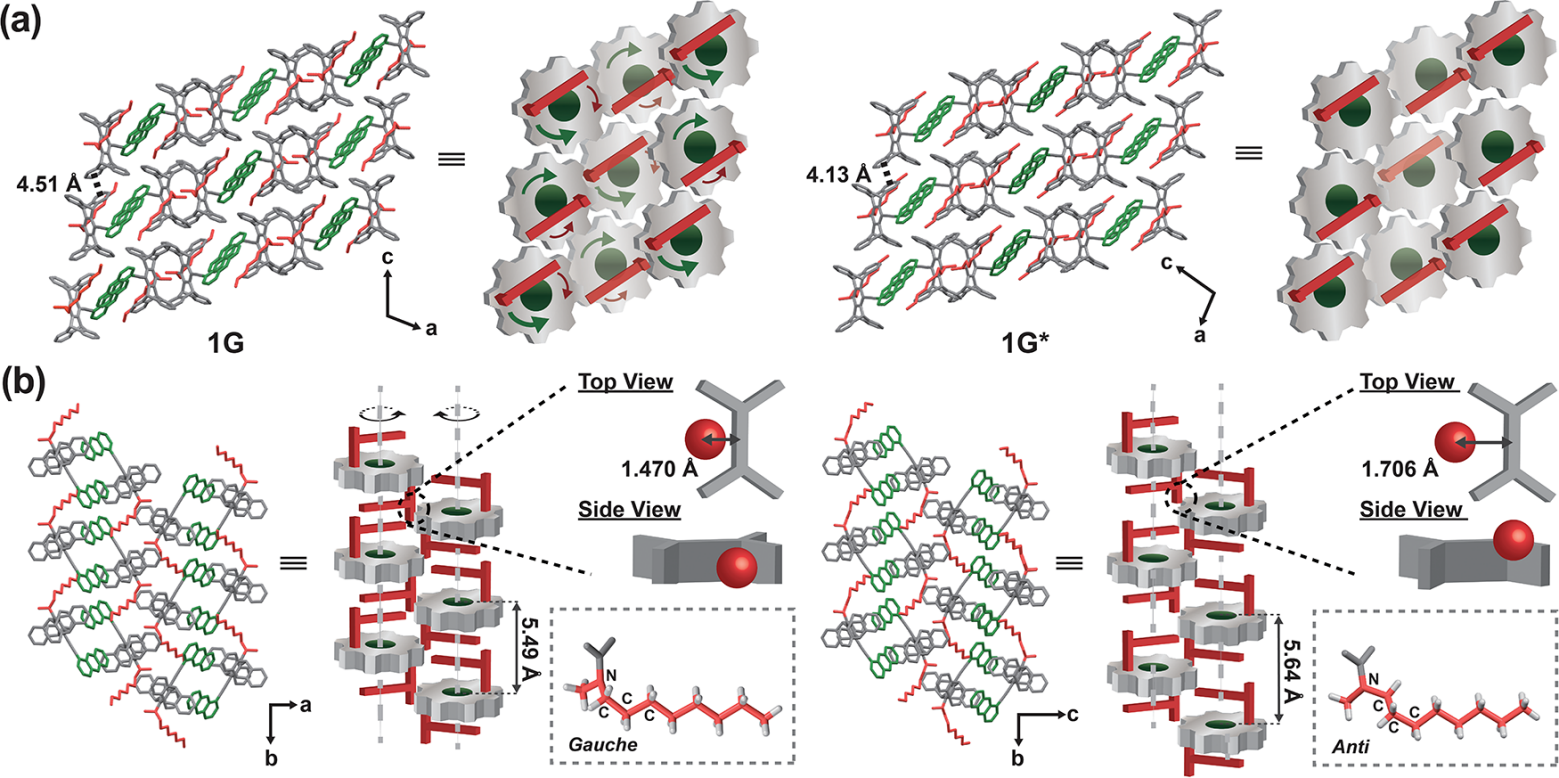
Comparison of crystal structures of **1G** (left) and 1G* (right): (a) (010) plane showing the pentiptycene V–V meshing and (b) gear network and schematic drawing of the octyl conformation and the position of the rotatable red tooth (the CH_3_–N–CH_2_ moiety) in the pentiptycene U-shaped cavity.

We propose that the 3D gear network in **1G** is crucial for molecular motion (i.e., gear rotation) and thus the observed phase transition. Accordingly, the gear disengagement in **1Y** might account for the irreversibility of the **1G**-to-**1Y** transition. By the same token, the distinct behavior for 1G* relative to **1G**, either the irreversibility of the **1G**-to-1G* transition or the inhibition of the 1G*-to-**1Y** transformation, might also be related to the change in 3D gear network, even though 1G* adopts a crystal packing mode similar to **1G**. Indeed, crucial differences in spatial relationships between adjacent supramolecular dimers in **1G** and 1G* were observed, as depicted in Figure 5 for comparison. While the meshing between adjacent pentiptycene V-shaped notches (the V–V meshing) along the *c* axis remained engaged, as indicated by the decreased π–π distance on going from **1G** (4.51 Å) to 1G* (4.13 Å), the position of the red teeth in 1G* slightly shifted out of the adjacent pentiptycene U-shaped cavity. This structural change was accompanied by the octyl conformation shifting from a gauche form to an *anti* form for the N–C–C–C moiety. Notably, the overall crystal density decreased when transitioning from **1G** (1.221 mg/m^3^) to 1G* (1.208 mg/m^3^). Evidently, solid-state molecular motion strongly depends on the crystal structure, and even a minor variation in the crystal structure can either enable or inhibit a specific molecular motion completely.

**Figure 6:**
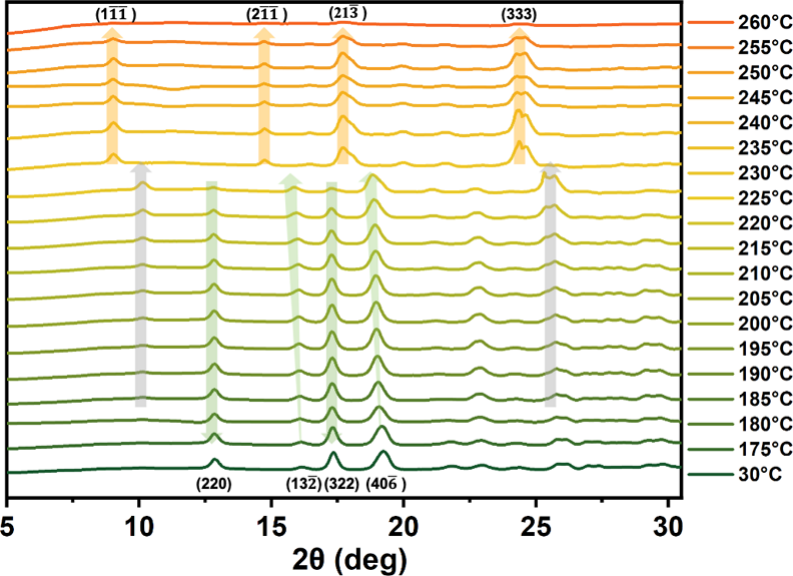
Variable-temperature synchrotron XRD measurements on the **1G**-to-**1Y** transformation (λ = 1.033 Å).

**An Intermediate State.** The variable-temperature synchrotron X-ray diffraction (VT XRD) results reveal the existence of an intermediate state, **1I**, during the **1G**-to-**1Y** transition (Figure 6). Upon heating **1G** to 180 °C, the signals at 2θ = 12.7° and 17.3°, corresponding to the (220) and (322) planes, begin to fade, while the signals at 2θ = 16.1° and 19.1°, associated with the (132̅) and (406̅) planes, appear to shift negatively without a decrease in intensity. This phenomenon could result from two neighboring signals, with one diminishing and the other intensifying. Notably, the (220) and (322) planes indicate intracolumnar molecular arrangements, while the (132̅) and (406̅) planes represent intercolumnar molecular arrangements (Figure S11). All four **1G** signals vanish at 230 °C, coinciding with the emergence of four new signals at 2θ = 8.9°, 14.6°, 17.7° and 24.3°, corresponding to the planes of (11̅1̅), (21̅1̅), (213̅), and (333) of **1Y**. These signals are related to intracolumnar molecular arrangements (Figure S12), reflecting the robust π_a_–π_p_ and π_a_–π_a_ interactions (Figure 1b). Moreover, noticeable signal changes occur in the temperature range of 180–230 °C, with a set of signals at 2θ = 10.1° and 25.3° increasing in intensity during this period. These signals vanish simultaneously with the aforementioned four **1G** signals at 230 °C. These two intermediate signals, along with the two signals near the **1G** peaks at 2θ = 16.1° and 19.1°, can be attributed to **1I**. Intriguingly, these four **1I** signals are in close proximity to the corresponding four **1Y** signals. Assuming a correlation between them, there exists molecular order in the intracolumnar relationship within **1I**. This conforms to the scenario of geared molecular rotation depicted in Figure 4c. Importantly, these VT XRD signals indicate that **1I** is not a molten or amorphous state.^71^

**Figure 7:**
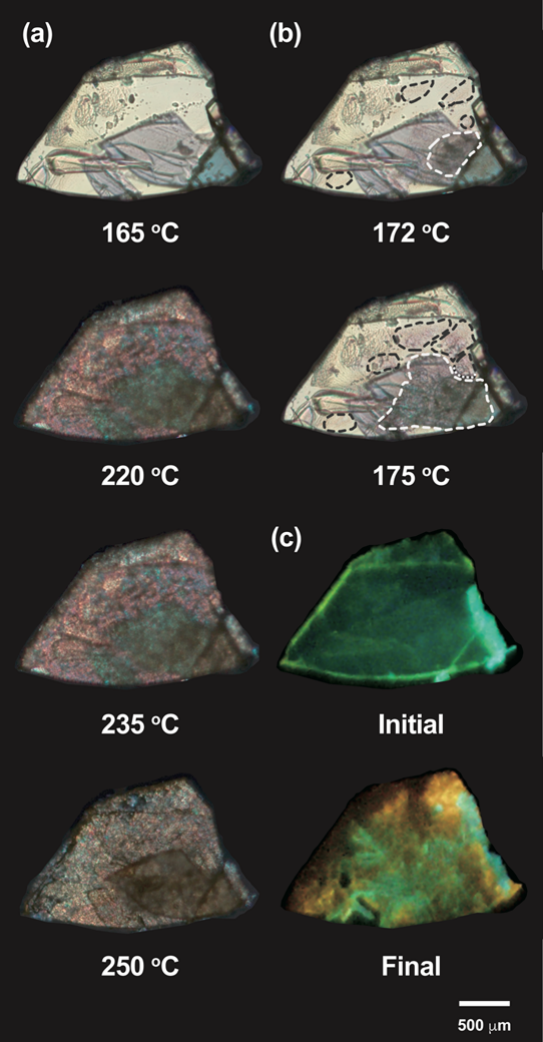
(a) Snapshots of POM images of a single crystal of **1G** during the heating process between 165 and 250 °C (heating rate: 10 °C/min); (b) POM images taken at 172 and 175 °C, emphasizing the radial propagation of birefringence (area outlined by dashed lines); (c) initial and final fluorescence images of the same crystal.

The hot-stage polarized optical microscopy (POM) measurement of a millimeter-sized single crystal of **1G** revealed a dynamic evolution of birefringence throughout the entire heating process from 165 to 250 °C (Movie S1). As illustrated in Figure 7a, birefringence changes commenced at 170 °C and encompassed the entire crystal at around 220 °C. Evidently, the entire crystal needed to be “activated” (around 220 °C) before the phase transition occurred (near 230 °C). These observations confirm the presence of not only a **1I** state but also crystallinity in the **1I** state. Furthermore, birefringence formation of the **1I** state exhibited a radial propagation pattern, as highlighted in Figure 7b. This characteristic aligns with the scenario of thermo-induced correlated molecular motions: once rotation initiates in a specific spot, neighboring molecules around the spot are simultaneously activated and then propagate radially. The fluorescence images of the same crystal before and after heating are shown in Figure 7c to show the bifurcated polymorphic transition.

The bifurcation of the polymorphic transition for **1G** indicates that the **1I** state structurally lies midway between **1Y** and 1G*. The scanning electron microscope (SEM) images of **1G**, **1Y**, and **1I** in polycrystalline powders show morphological changes on going from **1G** to **1Y** with the morphology of **1I** resembling **1G** more than that of **1Y** (Figure S13). Based on the 3D gear model, an oscillatory gear rotation state of the gears (i.e., the supramolecular dimers) within angles maintaining the gear network (i.e., less than 45° according to Figure 4e) can explain the **1I** state. Cooling from **1I** (170–230 °C) can recover **1G** (Figure S8), but its irreversible transition to **1Y** or 1G* at 230–235 °C depends on the direction of rotation. In **1I**, backward rotation forming 1G* follows the track of the 3D gear rotation, although a small structural change at the end of the rotation impairs the 3D gear network. However, the forward rotation forming **1Y** encounters significant conformational changes as well as a disengagement of the gear network at 45°–60° (Figure 4e), suggesting a higher energy barrier. It is known that crystal reconstruction is comparatively easier for smaller crystals due to their larger surface-to-volume ratio^72−74^ and for crystal with more defect sites.^54^ This same principle might explain the variation in the bifurcated polymorphic transition of **1G** observed in polycrystalline powders versus millimeter-sized crystals (Figure 2). Increasing the surface-to-volume ratio or the number of defect sites in crystals facilitates large molecular conformational changes toward **1Y**. Conversely, the opposite holds true for the formation of 1G*. Therefore, the relative fraction of **1Y** and 1G* would depend on both the size and the quality of individual crystals. In other words, at the phase-transition temperature, the formation of **1Y** is thermodynamically more favorable, while that of 1G* is kinetically more favorable.

**Recovery of 1G.** Despite the irreversible thermal phase transitions, **1G** can be restored through DCM vapor-fuming (Figure S5) or mechanical grinding (Figure S14) of **1Y** or 1G*. It is important to note that the identity of the green-emissive powders, whether **1G** or 1G*, can be readily determined based on their ability to transform into **1Y** upon heating to 245 °C (vide supra). Since **1G** is a crystallization-generated polymorph, the DCM vapor-induced **1Y**-to-**1G** or 1G*-to-**1G** conversion is consistent with the general concept that vapor fuming inherently involves recrystallization of the surface molecular layers.^7,8,40^ Notably, organic vapor-fuming is commonly employed to reverse phases induced by mechanical grinding.^7,8,41,60^ The shared **1Y**-to-**1G** phase transition induced by these two types of stimulation provides a rare example of convergent luminescence mechanochromism and vapochromism.

**Figure 8:**
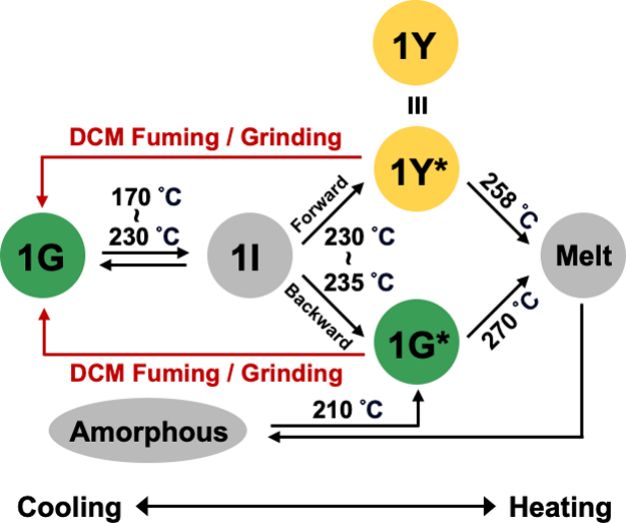
Summary of the phase transition behavior of **1**. The three polymorphs **1G**, **1Y**, and 1G* are colored according to their emission color, while the intermediate (**1I**), melt, and amorphous states are represented in gray.

Figure 8 summarizes the phase transition behavior of **1**. In brief, the phase transition of **1G** first undergoes a reversible process in the temperature range of 170–230 °C, forming an intermediate state, **1I**, followed by a concomitant and irreversible formation of two polymorphs, **1Y** and 1G*, at 230–235 °C, and then melt at ∼258 and ∼270 °C, respectively. The **1I** state represents an activation of the 3D gear rotation, and a disengagement of the gear network occurs upon forming **1Y** (a net 90° forward rotation) and 1G*. Polymorph 1G* is similar to **1G** but loses the ability of performing the gear rotation, hence cannot transform into **1Y** nor revert to **1G**. The cooling of the molten state generates an amorphous state, exclusively forming 1G* upon annealing near 210 °C. The recovery of **1G** can be achieved via grinding or DCM vapor-fuming of either **1Y** or 1G*.

**Mechanistic Features.** In principle, a polymorphic transition based on a 3D gear rotation corresponds to a cooperative (martensitic) transition mechanism. Our system indeed conforms to a typical first-order martensitic transition,^46^ where the generation of **1I** over a broad temperature range (170–230 °C) indicates a first-order nucleation/initiation stage. The subsequent propagation stage, leading to the formation of **1Y** and 1G* within a narrow temperature window (230–235 °C), aligns with cooperative molecular motions. This two-stage kinematic behavior resembles the mechanism observed in thermosalient crystals.^75^ However, no thermosalient effect was observed for the polymorphic transitions of **1G**. This observation might indicate the high adaptability of the crystal in dissipating strain, consistent with the high crystallinity and integrity of the daughter crystal. Notably, the irreversibility of the polymorphic transitions and the significant conformational and packing changes in the **1G**-to-**1Y** transformation deviate from the conventional scenario of cooperative transitions. Moreover, the bifurcation of the phase transition due to cooperative molecular motions has not been reported. These observations underscore the unique feature of the 3D gear rotation in polymorphic transitions.

Molecular gears play a vital role in the fields of molecular machines and nanotechnology.^76−78^ Considerable efforts have been dedicated to the development of rapid rotation or correlated motion of molecular components in crystals,^79−85^ although progress toward systems of 3D gear rotation is still in its infancy. In this context, the self-assembled 3D gear network in **1G** is fascinating. The **1G**-to-**1Y** transition beautifully illustrates how a 3D molecular gear system transfers the thermal energy from one shaft to another via rotational motion, inducing optical signal switching.

## Conclusions

In summary, we have elucidated the mechanistic aspects of molecular motion underlying the bifurcated polymorphic transitions from **1G** to **1Y** and 1G* along with concomitant thermochromic fluorescence. Notably, a bifurcated polymorphic transition of molecular crystals, to the best of our knowledge, is unprecedented. The *syn*-to-*anti* conformational shift, the constriction of supramolecular columns, and the transformation from monoclinic to triclinic crystal symmetry in the **1G**-to-**1Y** transition all stem from collective 3D gear rotation of the supramolecular dimers and columns. The 3D gear model is substantiated not only by the structural correlation between the mother (**1G**) and daughter (**1Y** and 1G*) phases but also by several key experimental observations. First, the polymorphic transition does not occur until the entire crystal is transformed into a dynamic intermediate state (**1I**), representing the activated form of 3D gear rotation. Second, the bifurcated transition of **1I** is explicable by the two potential outcomes of geared molecular rotation: the forward or backward rotation of the supramolecular columns in **1I**. Third, despite the structural similarities between **1G** and 1G*, the inability of 1G* to undergo the polymorphic transition to **1Y** emphasizes the essential role of the 3D gear network in driving the phase transition. This also explains the irreversibility of the enantiotropic transitions given that the 3D gear network is disengaged in both **1Y** and 1G*. Nevertheless, **1G** can be recovered by grinding or DCM vapor-fuming **1Y** or 1G*, enabling switchable fluorochromism. This work demonstrates the feasibility of constructing 3D molecular gear systems and underscores the intimate correlation between solid-state molecular motion and crystal packing. It highlights the boundless potential of crystal engineering in creating novel stimuli-responsive molecular crystals.

## Supporting Information Available

Experimental details and methods, emission spectra, crystal structures and information, NMR spectra and additional data (PDF)
Hot-stage POM of **1G** (AVI)

## Supplementary Material

ja3c12454_si_001.pdf

ja3c12454_si_002.avi
