# Supplementary material for: Bifurcated Polymorphic Transition and Thermochromic Fluorescence of a Molecular Crystal Involving Three-Dimensional Supramolecular Gear Rotation
Source: J Am Chem Soc. 2024 Mar 12;146(12):8131–41. doi: 10.1021/jacs.3c12454 (PMC10979455; doi:10.1021/jacs.3c12454)
Supplement: Supplementary file 1 — ja3c12454_si_001.pdf [file ja3c12454_si_001.pdf]

Supporting Information

Bifurcated Polymorphic Transition and Thermochromic Fluorescence of a Molecular Crystal Involving Three-Dimensional Supramolecular Gear Rotation

Yun-Hsuan Yang,† Yu-Shan Chen,† Wei-Tsung Chuang,‡ and Jye-Shane Yang†*

† Department of Chemistry, National Taiwan University, Taipei, Taiwan 10617

‡ National Synchrotron Radiation Research Center, Hsinchu, Taiwan 30092

Experimental Section...S4

General Methods. S4

Crystal Growth. S5

Stimulation-Induced Luminescence Responses. S5

Materials and Synthesis. S5

Scheme S1. Synthesis of compound **1**. S6

Tables...S8

Table S1. Photophysical data of **1** in dilute solutions (10^{-5} M) and in various solid-state forms. S8

Table S2. X-ray crystallographic data for the crystals of **1**. S9

Figures...S11

Figure S1. Normalized (a) absorption and (b) emission spectra and (c) fluorescence images of **1** in solutions (10 μM). S11

Figure S2. Nonbonded interactions in (a and c) **1G** and (b and d) **1Y**. In **1G**, adjacent A-pairs of supramolecular dimers within the same column exhibit octyl-anthracene C–H···π (CH_o-π_a) interactions, and the supramolecular columns are arranged side by side through *N*-methyl-pentiptycene C–H···π (CH_m-π_p) interactions. In **1Y**, the B-pairs exhibit anthracene-anthracene π_a-π_a interactions, and the inter-columnar interactions are *N*-octyl-pentiptycene C–H···π (CH_o-π_p) interactions. S12

Figure S3. X-ray crystal structure of **1Y** containing 30% of photodimer in the crystal: (a) crystal structure, in which the blue dots results from the photodimer; (b) the B-pair (top) and the resulting photodimer (bottom); (c) the supramolecular columns of monomer (top) and photodimer (bottom). S13

Figure S4. (a) ^{1}H-NMR spectrum of photodimer of **1** (400 MHz, $CDCl_3$); (b) $^{13}C\{^{1}H\}$-NMR spectrum of photodimer of **1** (100 MHz, $CDCl_3$).. S14

Figure S5. (a) PXRD patterns of compound **1**. The abbreviation for fuming DCM is “fum”, and for the simulated PXRD pattern form of single crystal is “simu”; (b) fluorescence images of compound **1** at different stages of stimuli response.. S15

Figure S6. Crystal of **1** (**1Y*** (left) and **1Y** (right)): (a) fluorescence images (scale bars = 200 μm) and the molecular conformation; (b) a ladder-like supramolecular column showing the slope, π_a-π_a interactions, and the π_a-π_p interactions; (c) a supramolecular sheet formed by the ladder-like supramolecular columns showing similar inter-columnar distances. Notice that the crystal structure of **1Y*** was determined from the yellow-emissive region of the thermally transformed crystal and that the crystal of **1Y** contains 30% photodimer......................... S16

Figure S7. Crystal of **1** (**1G** (left) and **1G*** (right)): (a) fluorescence images (scale bars = 200 μm) and the molecular conformation; (b) a ladder-like supramolecular column showing the slope, CH-π_a and the π_a-π_p interactions; (c) a supramolecular sheet formed by the ladder-like supramolecular columns showing similar inter-columnar distances. Notice that the crystal structure of **1G*** was determined from the green-emissive region of the thermally transformed crystal.. S17

Figure S8. DSC scans of **1G** at 2 °C /min: (first round heating-cooling cycle) heating terminated at 225 °C; (second round heating-cooling cycle) curves are essentially the same as curves 4 in Figure 3 (i.e., behaves like fresh **1G**); (third round heating-cooling cycle) curves are essentially the same as curves 5 in Figure 3. Values around the peak indicate the corresponding peak temperature (°C). S18

Figure S9. DSC scans of **1G** at different scan rates: (from top to bottom) scan rate = 15, 10, 5, and 2 °C/min. Values around the peak indicate the corresponding peak temperature (°C). S19

Figure S10. Void space (yellow area) in **1G**.. S20

Figure S11. The (220), (322), ($13\bar{2}$), and ($40\bar{6}$) planes of **1G** corresponding to the PXRD signals at 2θ = 12.7°, 17.3°, 16.1°, and 19.1°, respectively. S21

Figure S12. The ($1\bar{1}\bar{1}$), ($2\bar{1}\bar{1}$), ($21\bar{3}$), and (333) planes of **1Y** corresponding to the PXRD signals at 2θ = 8.9°, 14.6°, 17.7° and 24.3°, respectively. S22

Figure S13. Scanning electron microscope (SEM) images of **1G**, **1I**, and **1Y** (from top to bottom) in polycrystalline powders.. S23

Figure S14. Fluorescence images (scale bars: 200 μm) of mechanical grinding followed by heating to 245°C for (a) **1G**, (b) **1G***, and (c) **1Y**.. S24

NMR spectra..S25

Figure S15. ^{1}H-NMR spectrum of compound **1** (400 MHz, $CDCl_3$)................................ S25

Figure S16. ^{13}C{^{1}H}-NMR spectrum of compound **1** (100 MHz, $CDCl_3$). S25

Figure S17. ^{1}H-NMR spectrum of compound **3** (400 MHz, $CDCl_3$)................................ S26

Figure S18. ^{13}C{^{1}H}-NMR spectrum of compound **3** (100 MHz, $CDCl_3$). S26

Figure S19. ^{1}H-NMR spectrum of compound **4** (400 MHz, $CDCl_3$)................................ S27

Figure S20. ^{13}C{^{1}H}-NMR spectrum of compound **4** (100 MHz, $CDCl_3$). S27

Reference S28

Experimental Section.

General Methods.

The ^{1}**H NMR** spectra and ^{13}**C NMR** spectra were recorded by Bruker AVIII-400 MHz and chemical shifts (δ) were reported in parts per million (ppm) relative to $CDCl_3$ (1H: δ = 7.26, 13C: δ = 77.00). **High-resolution mass data** were collected by electrospray ionization (ESI) with a Bruker micro TOF-QII spectrometer. **UV/visible spectra** were recorded using a Cary 300 double-beam spectrophotometer. **Emission and excitation spectra** were recorded at ambient temperature by using an Edinburgh FLS920 spectrometer corrected for the R928P detector or by an Ocean USB2000 spectrometer. A solution of quinine bisulfate [Φ_f = 0.546 in 1N H_2SO_4(aq)] was used as the standard [S1] for the luminescence quantum yield determination of compounds in N_2-purged solutions. The optical density of all solutions was about 0.1 at the wavelength of excitation, and an error of 5% was estimated for emission quantum yields. The absolute luminescence quantum yields for the crystalline samples were determined using an integrating sphere (150 mm diameter, $BaSO_4$ coating) and the Edinburgh FLS920 spectrometer. The **luminescence lifetimes** were measured using an FLS980 spectrometer equipped with a 290 nm picosecond pulsed diode laser, and the signal was collected using a time-dependent single photon counting system (OB-900 L lifetime spectrometer, Edinburgh). The goodness of the nonlinear least-squares fit for phosphorescence was judged by the χ^2 value (0.8< χ^2 < 1.2). **Fourier transform infrared (FT-IR) spectra** were recorded on a Thermo Scientific Nicolet iS5 FT-IR spectrometer by using drop-casting method on a KBr pellet. **Luminescence images** were recorded by an Olympus IX73 inverted microscope equipped with a DP73 color camera. The light source of the microscope is a halogen lamp equipped with an Olympus U-FUW filter, which passed light in the range of 340–390 nm. **Powder X-ray diffraction (PXRD**) were measured by Bruker AXS D2 Phaser using nickel-filtered Cu Kα radiation (λ = 1.5418 Å) at a voltage of 30 mV and a current of 10 mA. **Crystal structures** were determined using a Rigaku Oxford Diffraction diffractometer (Xcalibur, Atlas, Gemini) or Bruker AXS D8 VENTURE. The simulated PXRD of the crystals were provided by the Mercury program. **Polarized Optical Microscopic images** were recorded by a LEICA DM 2500P inverted microscope equipped with a Linkam LNP96S hot stage. The light source for the microscope is a halogen lamp (100 W). Each image was taken using a charge-couple device mounted on the microscopic with an exposure time of 500 ms. The images with the crossed polarizers at 90° were captured by rotating the samples when the two crossed polarizers are fixed at 90°. **Differential scanning calorimetry** was performed on a premium differential scanning calorimeter, Netzsch 204 F1. **VT-XRD** measurement was performed at TLS 01C2 of

the National Synchrotron Radiation Research Center (NSRRC), Taiwan. The data were collected with 12keV X-ray of a wavelength of 1.033210 Å, whereas the data were collected with an image plate (Mar2400), and the scattering angle 2θ was calibrated with diffractions from silver behenate. The crystal **porosity** was analyzed by Solv. Plot analysis of PLATON software using the crystal structure data removing solvent molecules. **Scanning electron microscopy (SEM)** images were taken with a JEOL JSM-7600F field emission scanning electron microscope with INCA X-Max EDS.

Crystal Growth.

The growth of single crystals of **1** was carried out by the solvent layering method. The substrate (3 mg) was first dissolved in 1.8 mL of good solvent dichloromethane (DCM) in a vial, and a layer of poor solvent methanol (1.8 mL) was then carefully added on the top of the DCM solution. The vial was loosely capped and stored at ambient temperature for crystallization.

Stimulation-Induced Luminescence Responses.

For thermofluorochromic tests, the sample was placed on a quartz plate and then heated on a hot plate or a hot stage under nitrogen in the dark at a heating rate of 10°C/min, after reaching 245°C, the sample was heated for 15 minutes. For vapofluorochromic tests, the sample was placed on a quartz plate and was placed in a 20 mL vial bottle that contained a piece of cotton soaked with 0.5 mL of DCM solvents for 20 min. For the mechanofluorochromic tests, the samples on a quartz plate was carried out by gentle hand grinding with a spatula for 8 min, when the luminescence color was uniform and no longer changed (in the case of **1Y**-to-**1G** transition). The same grinding time were applied to the cases of **1G** and **1G***.

Materials and Synthesis.

All commercial reagents, catalysts, and solvents (HPLC grade for photophysical measurements) were used as received. Column chromatography was carried out on silica gel (Geduran® SI 60). The syntheses of compound 1 are shown in Scheme S1, in which the starting material 2 [S2] were synthesized according to the literature procedure, followed by the synthetic procedures and compound characterization data.

Scheme S1. Synthesis of compound **1.**

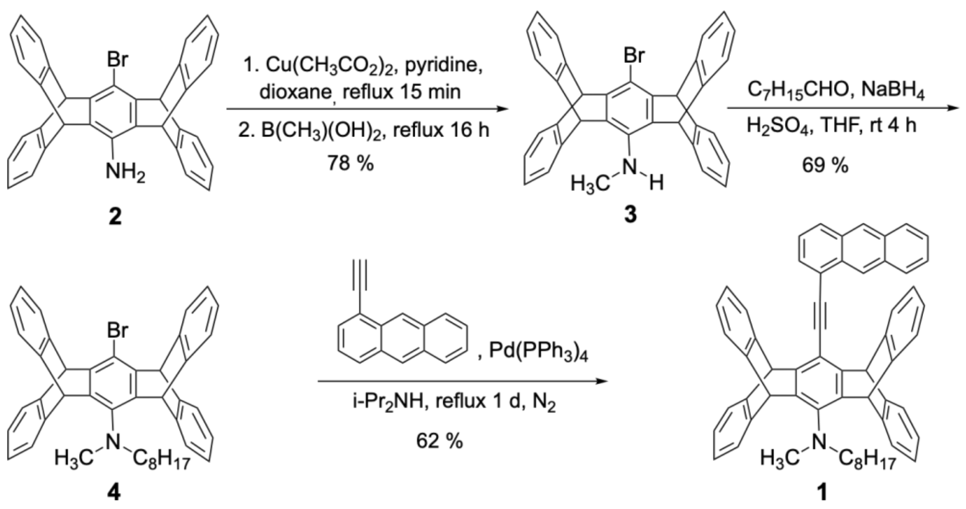

Synthesis of compound 1. Under a nitrogen atmosphere, a mixture of **3** (100 mg, 0.15 mmol), **4** (28.0 mg, 0.14 mmol), and $Pd(PPh_3)_4$ (16.0 mg, 0.01 mmol) was placed in a 25 mL two-neck round-bottom flask containing 12 mL of degassed diisopropylamine. The mixture was refluxed at 90°C for 24 hours. After cooling to room temperature, the solvent was removed under reduced pressure, and the residue was filtered through Celite with DCM. The resulting crude product was purified by column chromatography using DCM/hexane (1:8, v/v) as the eluent to afford the yellow solid **1** (73.6 mg, 62% yield); mp: 260.5-261.1°C, ^{1}H NMR (400 MHz, $CDCl_3$): δ = 0.90 (t, *J* = 6.9 Hz, 3H), 1.29-1.41 (m, 12H), 3.07 (s, 3H), 3.38 (t, *J* = 7.1 Hz, 2H), 5.87 (s, 2H), 6.06 (s, 2H), 6.95-6.97 (m, 8H), 7.34-7.39 (m, 4H), 7.41-7.42 (m, 4H), 7.56-7.63 (m, 3H), 8.02 (d, *J* =7.1 Hz, 1H), 8.10-8.18 (m, 3H), 8.58 (s, 1H), 9.27 (s, 1H) **(Figure S15)**; $^{13}C\{^1H\}$ NMR (100 MHz, $CDCl_3$): δ = 14.1, 22.7, 27.4, 29.4, 29.6, 29.7, 31.9, 43.1, 50.1, 52.7, 57.7, 90.7, 93.3, 112.5, 121.6, 123.4, 123.9, 124.9, 125.0, 125.2 (2C), 126.0, 126.1, 127.2, 128.2, 128.5, 129.3, 130.2, 131.4, 132.1, 132.4, 141.5, 143.4, 145.2 (2C), 145.3, 145.4 **(Figure S16)**; IR (KBr): 527, 555, 564, 649, 703, 730, 753, 792, 879, 1022, 1108, 1150, 1168, 1196, 1263, 1316, 1378, 1460, 1477, 1613, 2204, 2853, 2925, 3020, 3066 cm^{-1}; HRMS (ESI-TOF): m/z calculated for $C_{59}H_{50}N$ ($[M+H]^+$): 772.3938, found: 772.3931.

Synthesis of compound 3. Under a nitrogen atmosphere, a mixture of **3** (500 mg, 0.95 mmol) and copper acetate (415 mg, 2.28 mmol) was prepared in 40 mL of degassed 1,4-dioxane, along with 0.28 mL of pyridine (3.47 mmol), in a two-neck round-bottom flask. The mixture was refluxed at 110°C for 15 minutes, and then methylboronic acid (142.5 mg, 2.38 mmol) was added. The reaction was refluxed for an additional 16 hours. After cooling to room temperature, the solvent was removed under reduced pressure,

and the residue was dissolved in DCM and washed with ammonia and brine, respectively. The organic layer was dried over anhydrous $MgSO_4$, and the filtrate was concentrated under reduced pressure. The resulting crude product was purified by column chromatography using EA/DCM/hexane (1:1:5, v/v) as the eluent, yielding white solid **3** (0.4 g, 78% yield); mp: >300°C, ^{1}H NMR (400 MHz, $CDCl_3$): δ = 2.93 (s, 3H), 5.66 (s, 2H), 5.89 (s, 2H), 6.95-6.99 (m, 8H), 7.34-7.36 (m, 4H), 7.38-7.39 (m, 4H) **(Figure S17)**; ^{13}C{^{1}H} NMR (100 MHz, $CDCl_3$): δ = 38.4, 49.6, 53.7, 111.7, 123.3, 123.9, 125.3 (2C), 138.7, 139.6, 142.5, 144.9, 145.1 **(Figure S18)**; IR (KBr): 541, 572, 597, 646, 667, 745, 1179, 1458, 1634, 2980, 3071, 3367 cm^{-1}; HRMS (ESI-TOF): m/z calculated for $C_{35}H_{25}BrN$ ($[M+H]^+$): 538.1165, found: 538.1166.

Synthesis of compound 4. Compound **3** (200 mg, 0.37 mmol) was dissolved in 6 mL of tetrahydrofuran, and then octanal (63.8 μL, 0.41 mmol) and 0.3 mL of 3 M sulfuric acid were added to the round-bottom flask. The mixture was stirred at room temperature for 5 minutes, and then sodium borohydride (15.5 mg, 0.41 mmol) was added. After the reaction proceeded for 4 hours, water was added and the mixture was stirred for 10 minutes. The solvent was removed under reduced pressure, and the residue was dissolved in DCM and washed with brine. The organic layer was dried over anhydrous $MgSO_4$, and the filtrate was concentrated under reduced pressure. The resulting crude product was purified by column chromatography using DCM/hexane (1:3, v/v) as the eluent, yielding white solid **4** (166 mg, 69% yield); mp: 261.6-263.4°C, ^{1}H NMR (400 MHz, $CDCl_3$): δ = 0.89 (t, J = 6.9 Hz, 3H), 1.26-1.35 (m, 10H), 1.47-1.51 (m, 2H), 2.99 (s, 3H), 3.30 (t, J = 7.4 Hz, 2H), 5.82 (s, 2H), 5.85 (s, 2H), 6.91-6.97 (m, 8H), 7.30 (s, 4H), 7.35-7.37 (m, 4H) **(Figure S19)**; ^{13}C{^{1}H} NMR (100 MHz, $CDCl_3$): δ = 14.1, 22.7, 27.4, 29.3, 29.6, 29.7, 31.9, 43.1, 50.3, 53.7, 57.7, 113.9, 123.4, 123.9, 125.2, 142.0, 142.6, 143.8, 144.9, 145.1, 145.2 **(Figure S20)**; IR (KBr): 540, 585, 614, 648, 675, 703, 742, 752, 885, 1023, 1108, 1155, 1178, 1196, 1264, 1298, 1376, 1435, 1459, 1633, 1658, 2851, 2922, 3020, 3041, 3069 cm^{-1}; HRMS (ESI-TOF): m/z calculated for $C_{43}H_{41}BrN$ ($[M+H]^+$): 650.2417, found: 650.2437.

Tables

Table S1. Photophysical data of **1** in dilute solutions (10^{-5} M) and in various solid-state forms.

Form	λ_{ab} (nm)[a]	λ_f (nm)[b]	Φ_f[c]	τ_1 (ns)[c]	τ_2 (ns)[c]
Hexane	378 (398)	437	0.19	3.15	-
THF	381 (401)	502	0.36	4.24	-
DCM	381 (400)	511	0.31	4.32	-
CH_3CN	383 (402)	574	0.50	6.39	-
$1G_{powder}$	-	541	0.74	2.45 (65%)	5.79 (35%)
$1Y_{powder}$	-	596	0.24	38.77 (61%)	5.19 (39%)
$1G_{crystal}$	-	541	0.77	3.45 (100%)	-
$1Y_{crystal}$	-	587	0.59	37.34 (91%)	3.05 (9%)

[a] The parentheses are secondary absorption bands. [b] λ_{ex}= 380 nm. [c] λ_{ex}= 375 nm.

Table S2. X-ray crystallographic data for the crystals of **1**.

**Crystal**	**1G**	**1G***
Empirical formula	$C_{59}H_{49}N$	$C_{59}H_{49}N$
Formula weight	771.99	771.99
Crystal system	Monoclinic	Monoclinic
Space group	$P2_1/c$	$P2_1/c$
Crystal size(mm^3)	0.250 × 0.200 × 0.150	0.250 × 0.200 × 0.150
a (Å)	15.4624(13)	11.7571(2)
b (Å)	11.8288(12)	12.5138(3)
c (Å)	23.834(3)	30.0568(6)
α (°)	90	90
β (°)	105.495(10)	106.3240(9)
γ (°)	90	90
V (Å^3)	4200.8(8)	4243.87(15)
Z value	4	4
F(000)	1640	1640
D_{cal} (Mg/m^{-3})	1.221	1.208
Wavelength (Å)	0.71073	1.54178
Cell parameters reflection used	6625	9963
Theta range for Cell parameters	3.7380 to 30.0500°	3.06 to 78.34°
Absorption coefficient (mm^{-1})	0.069	0.519
Temperature (K)	120(2)	100(2)
Diffractometer	Xcalibur, Atlas, Gemini	Bruker AXS D8 VENTURE
Absorption correction	Semi-empirical from equivalents	Semi-empirical from equivalents
Max. and min. transmission	1.00000 and 0.95940	1.0000 and 0.9133
No. of measured reflections	26846	82418
No. of independent reflections	9635 [R(int) = 0.0542]	8824 [R(int) = 0.0354]
No. of observed [I>2_igma(I)]	6210	8132
Completeness to theta = 24.999°	99.8%	-
Completeness to theta = 67.679°	-	99.5%
Theta range for data collection	3.152 to 27.499°	3.064 to 78.534°
Final R indices [I>2sigma(I)]	R1 = 0.0571, wR2 = 0.1241	R1=0.0464,wR2=0.1251
R indices (all data)	R1 = 0.1006, wR2 = 0.1609	R1=0.0495,wR2=0.1284
Goodness-of-fit on F2	1.023	1.023
No. of reflections	9635	8824
No. of parameters	541	543
No. of restraints	0	0

Table S2 (continued). X-ray crystallographic data for the crystals of **1**.

**Crystal**	**1Y**	**1Y***
Empirical formula	$C_{59}H_{49}N$	$C_{59}H_{49}N$
Formula weight	771.99	771.99
Crystal system	Triclinic	Triclinic
Space group	$P\bar{1}$	$P\bar{1}$
Crystal size(mm^3)	0.180 × 0.120 × 0.030	0.250 × 0.200 × 0.100
a (Å)	9.1337(3)	9.2775(4)
b (Å)	11.6033(4)	11.7412(5)
c (Å)	19.8199(6)	19.9994(8)
α (°)	81.2028(12)	80.7852(19)
β (°)	84.0601(13)	82.984(2)
γ (°)	86.5301(12)	85.756(2)
V (Å^3)	2062.54(12)	2131.00(16)
Z value	2	2
F(000)	820	820
D_{cal} (Mg/m^{-3})	1.203	1.203
Wavelength (Å)	1.54178	1.54178
Cell parameters reflection used	9913	9901
Theta range for Cell parameters	2.27 to 78.11°	2.25 to 74.45°
Absorption coefficient (mm^{-1})	0.534	0.517
Temperature (K)	100(2)	99(2)
Diffractometer	Bruker AXS D8 VENTURE	Bruker AXS D8 VENTURE
Absorption correction	Semi-empirical from equivalents	Semi-empirical from equivalents
Max. and min. transmission	1.0000 and 0.828156	1.00000 and 0.8905
No. of measured reflections	32470	39711
No. of independent reflections	8506 [R(int) = 0.413]	7711 [R(int) = 0.0462]
No. of observed [I>2_igma(I)]	7295	5806
Completeness to theta = 67.679°	99.5%	99.3%
Theta range for data collection	2.266 to 78.955°	2.252 to 67.994°
Final R indices [I>2sigma(I)]	R1=0.0450,wR2=0.1166	R1 = 0.0675,wR2 =0.2148
R indices (all data)	R1=0.0532,wR2=0.1240	R1 = 0.0873,wR2 =0.2330
Goodness-of-fit on F2	1.027	1.671
No. of reflections	8506	7711
No. of parameters	543	743
No. of restraints	0	1278

Figures

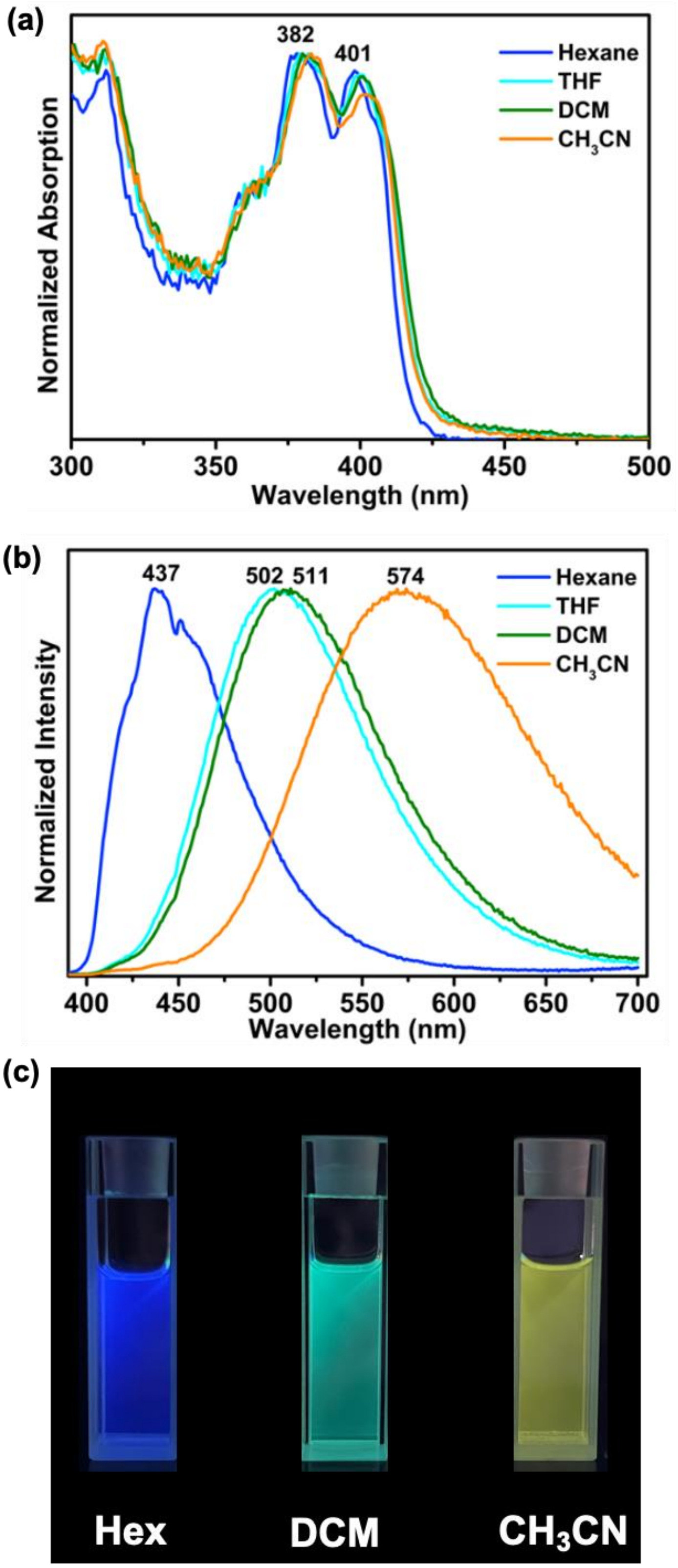

Figure S1. Normalized (a) absorption and (b) emission spectra and (c) fluorescence images of **1** in solutions (10 μM).

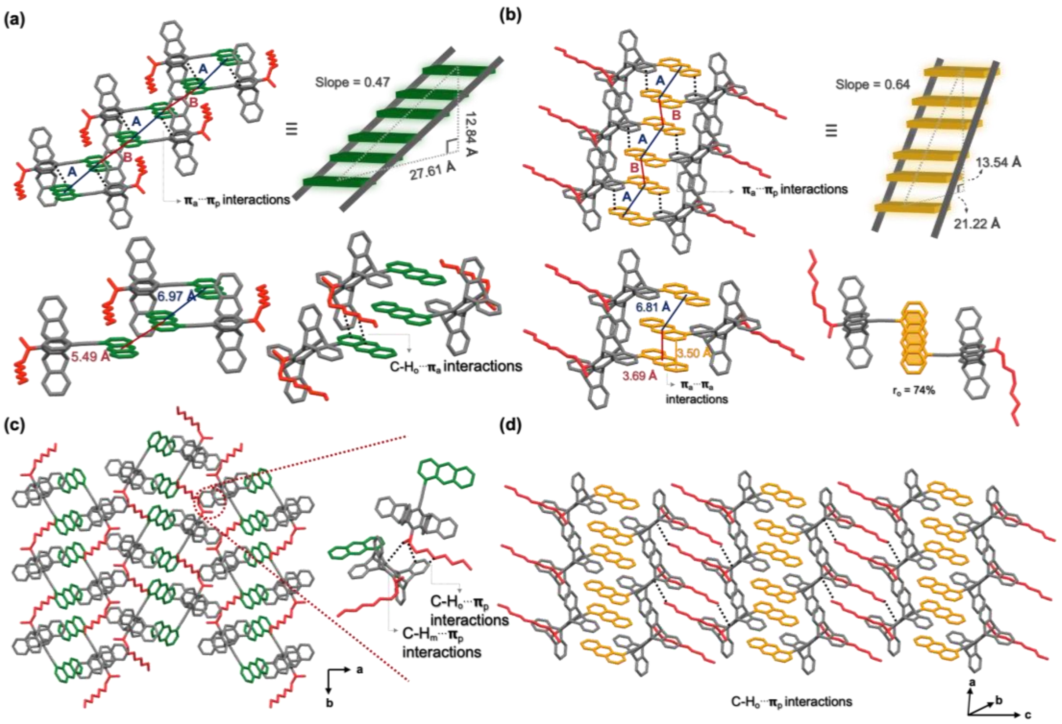

Figure S2. Nonbonded interactions in (a and c) **1G** and (b and d) **1Y**. In **1G**, adjacent A-pairs of supramolecular dimers within the same column exhibit octyl-anthracene C−H···π (CH_o-π_a) interactions, and the supramolecular columns are arranged side by side through *N*-methyl-pentiptycene C−H···π (CH_m-π_p) interactions. In **1Y**, the B-pairs exhibit anthracene-anthracene π_a-π_a interactions, and the inter-columnar interactions are *N*-octyl-pentiptycene C−H···π (CH_o-π_p) interactions.

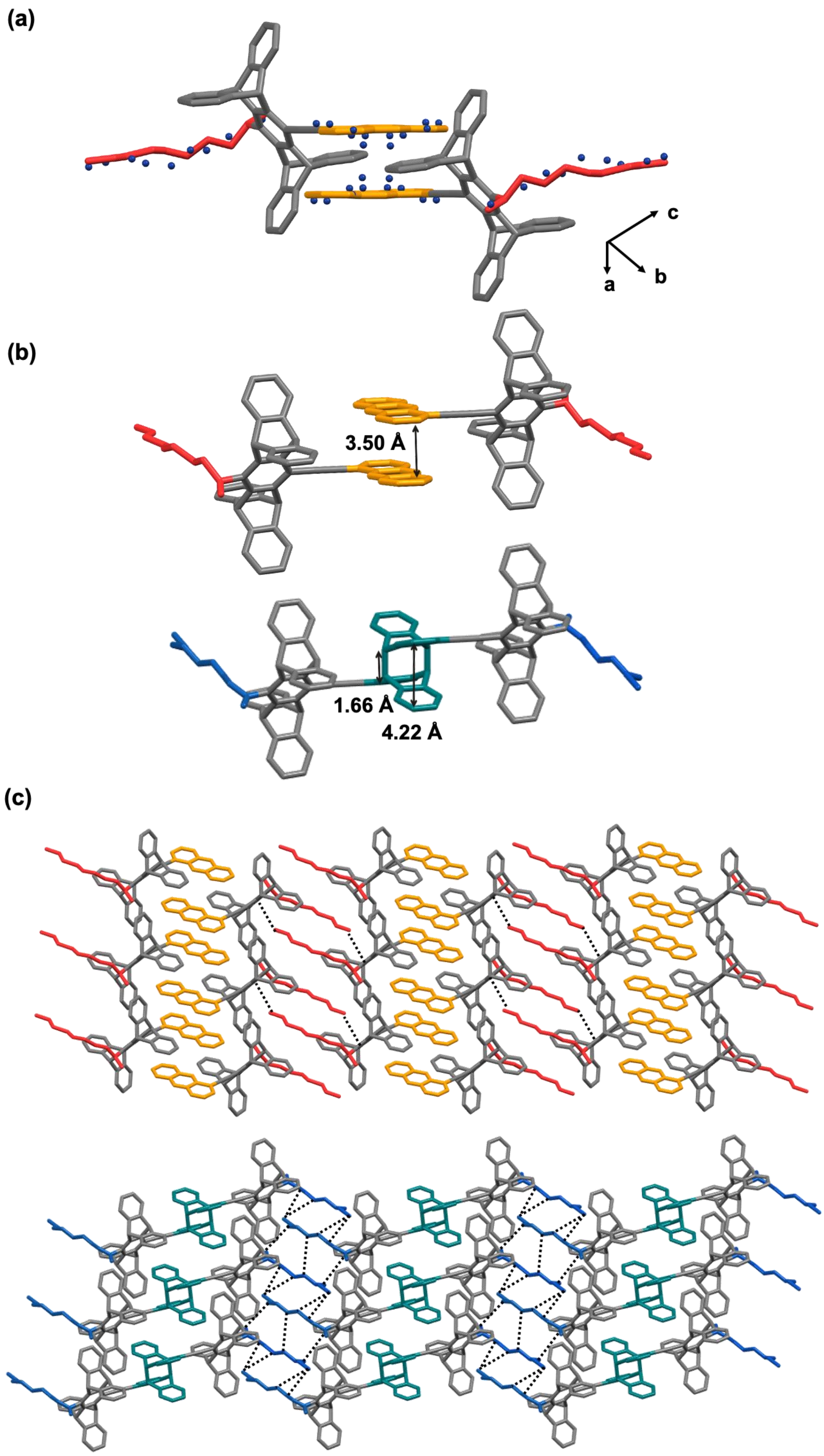

Figure S3. X-ray crystal structure of **1Y** containing 30% of photodimer in the crystal: (a) crystal structure, in which the blue dots results from the photodimer; (b) the B-pair (top) and the resulting photodimer (bottom); (c) the supramolecular columns of monomer (top) and photodimer (bottom).

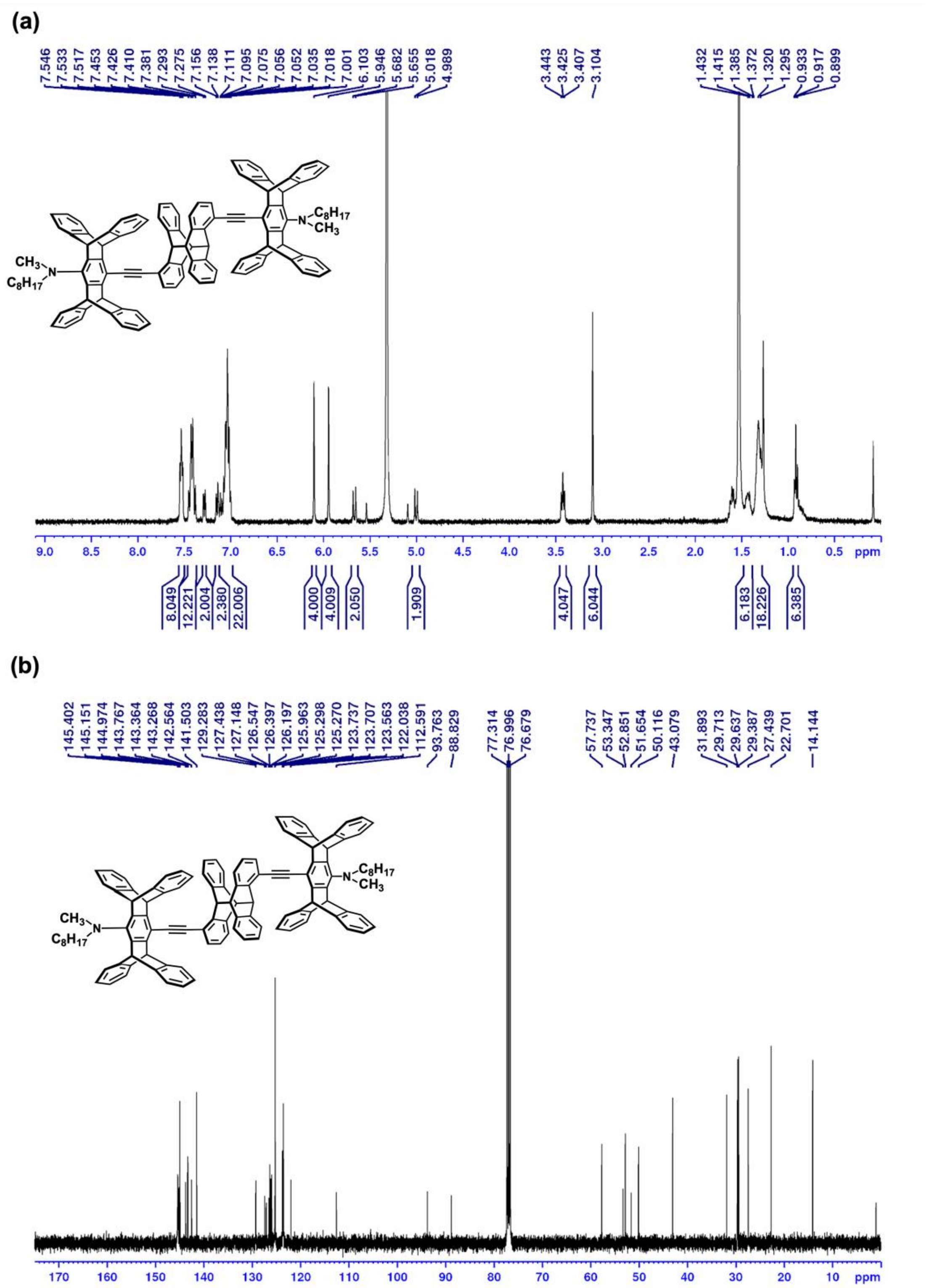

Figure S4. (a) ^{1}H-NMR spectrum of photodimer of **1** (400 MHz, $CDCl_3$); (b) $^{13}C\{^{1}H\}$-NMR spectrum of photodimer of **1** (100 MHz, $CDCl_3$).

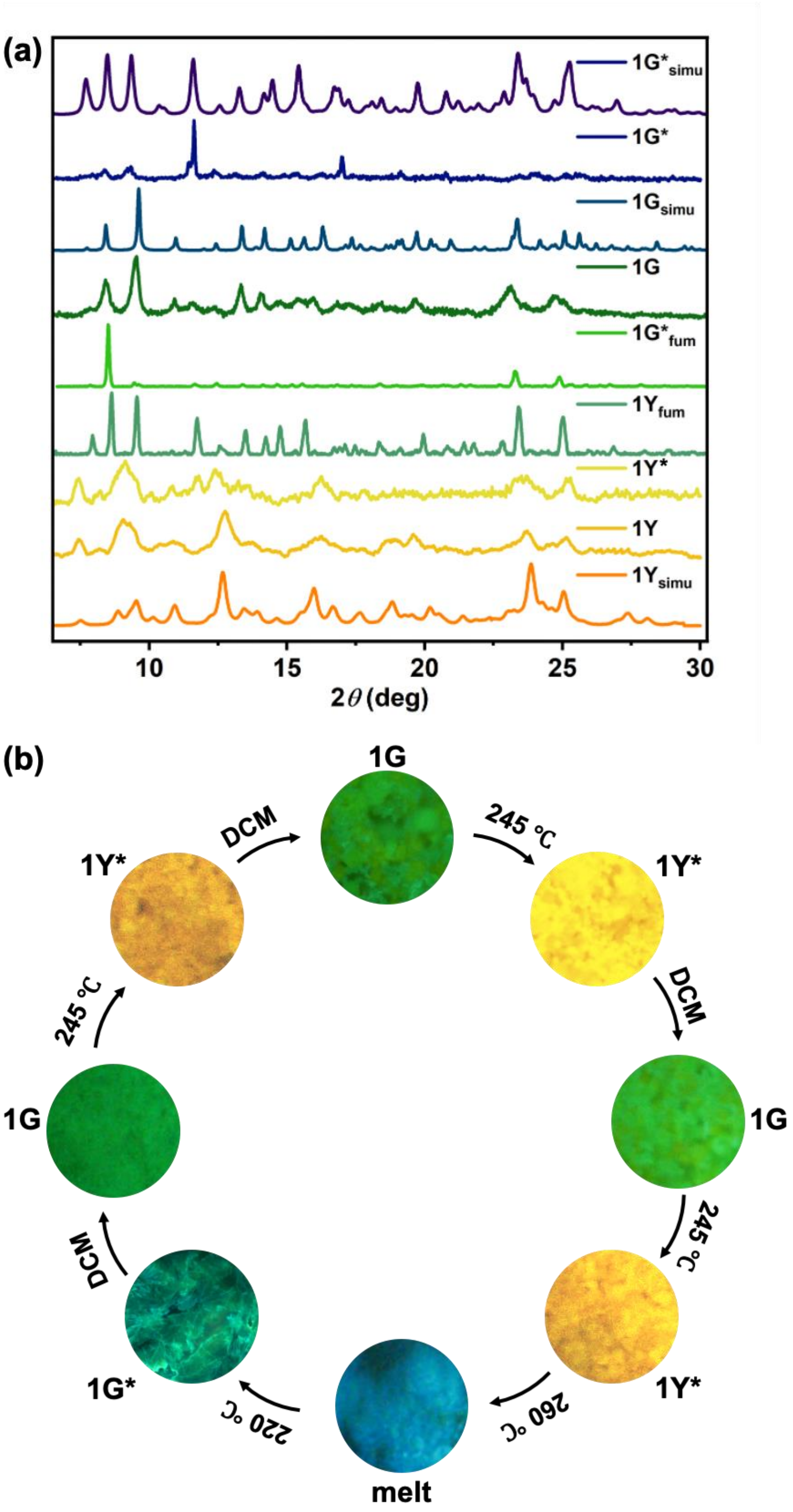

Figure S5. (a) PXRD patterns of compound **1**. The abbreviation for fuming DCM is "fum", and for the simulated PXRD pattern form of single crystal is "simu"; (b) fluorescence images of compound **1** at different stages of stimuli response.

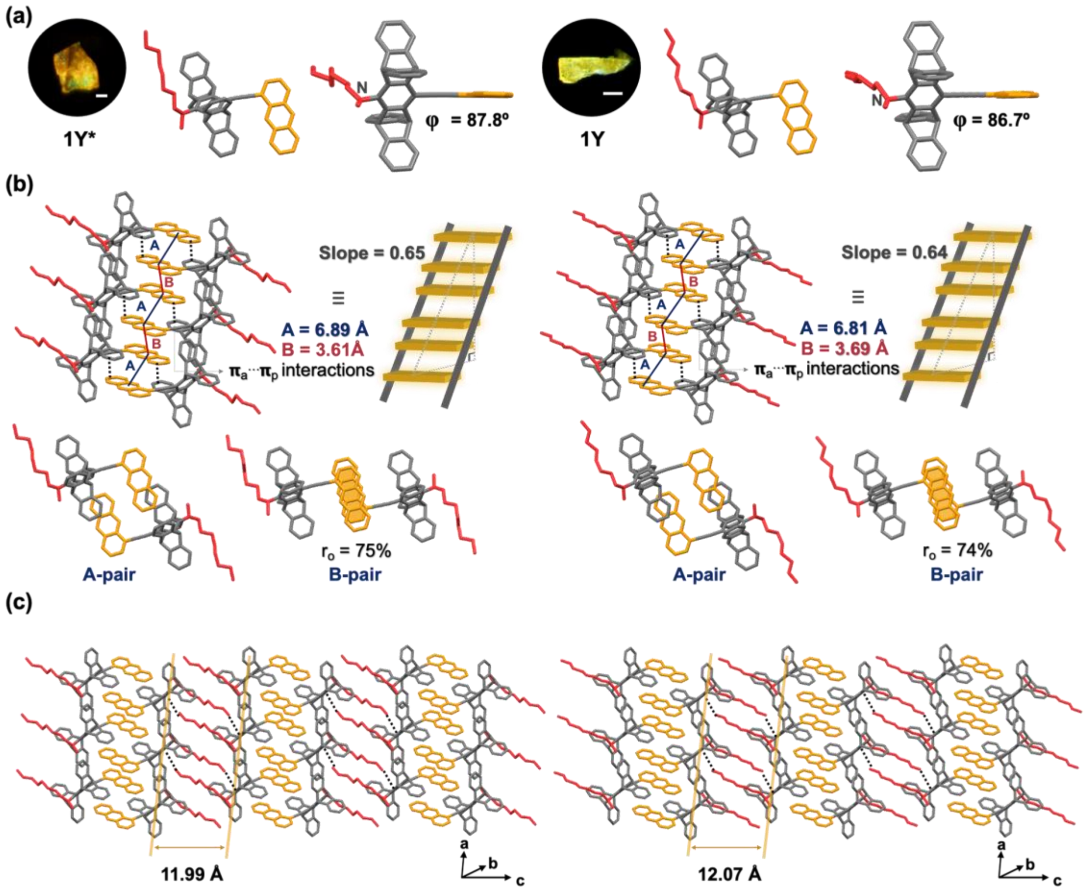

Figure S6. Crystal of **1** (**1Y*** (left) and **1Y** (right)): (a) fluorescence images (scale bars = 200 μm) and the molecular conformation; (b) a ladder-like supramolecular column showing the slope, π_a-π_a interactions, and the π_a-π_p interactions; (c) a supramolecular sheet formed by the ladder-like supramolecular columns showing similar inter-columnar distances. Notice that the crystal structure of **1Y*** was determined from the yellow-emissive region of the thermally transformed crystal and that the crystal of **1Y** contains 30% photodimer.

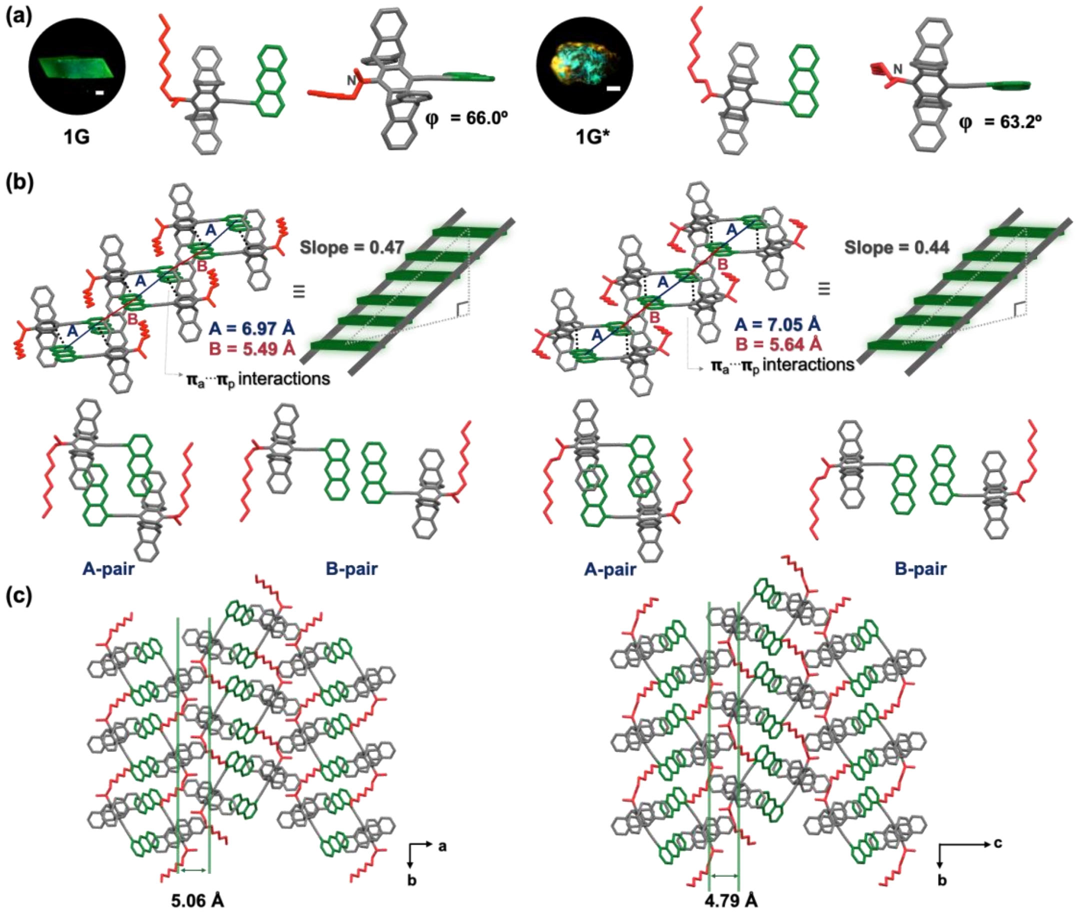

Figure S7. Crystal of **1** (**1G** (left) and **1G*** (right)): (a) fluorescence images (scale bars = 200 μm) and the molecular conformation; (b) a ladder-like supramolecular column showing the slope, CH-π_a and the π_a-π_p interactions; (c) a supramolecular sheet formed by the ladder-like supramolecular columns showing similar inter-columnar distances. Notice that the crystal structure of **1G*** was determined from the green-emissive region of the thermally transformed crystal.

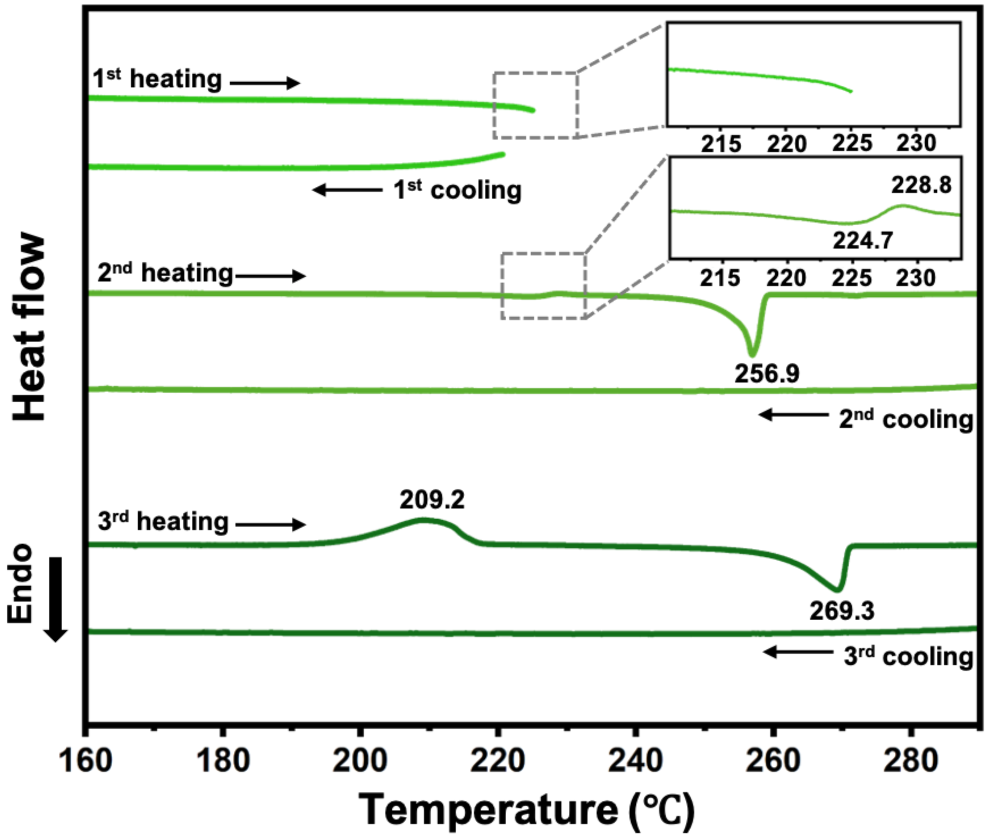

Figure S8. DSC scans of **1G** at 2 °C /min: (first round heating-cooling cycle) heating terminated at 225 ºC; (second round heating-cooling cycle) curves are essentially the same as curves 4 in Figure 3 (i.e., behaves like fresh **1G**); (third round heating-cooling cycle) curves are essentially the same as curves 5 in Figure 3. Values around the peak indicate the corresponding peak temperature (°C).

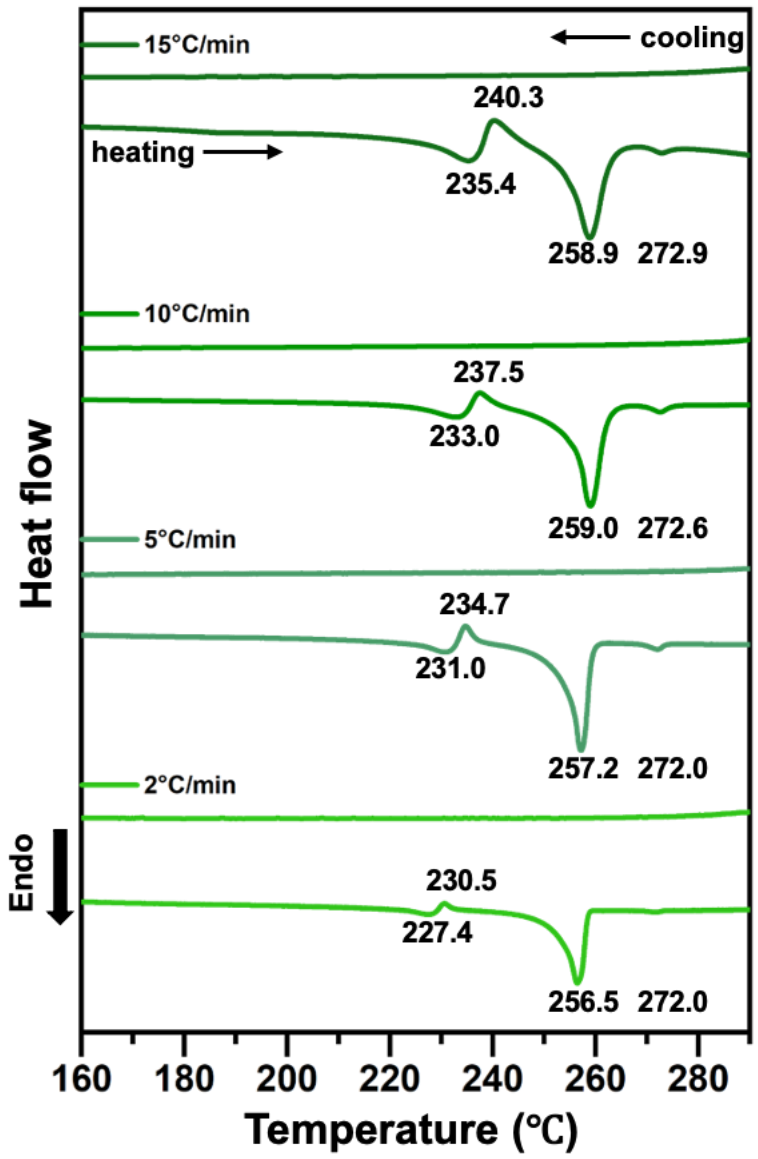

Figure S9. DSC scans of **1G** at different scan rates: (from top to bottom) scan rate = 15, 10, 5, and 2 °C/min. Values around the peak indicate the corresponding peak temperature (°C).

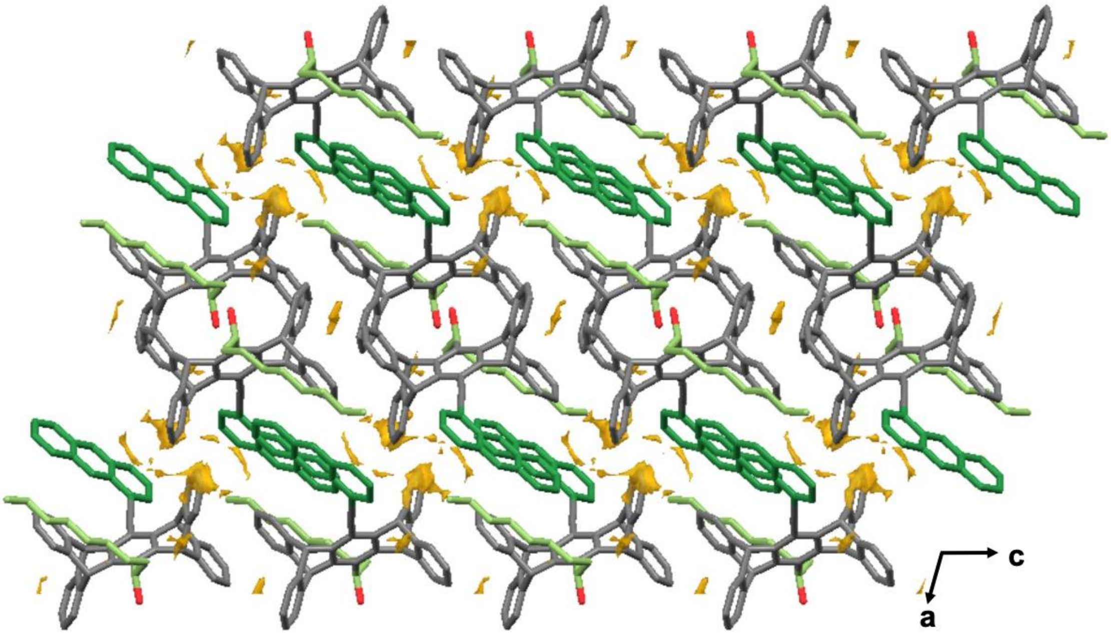

Figure S10. Void space (yellow area) in **1G**.

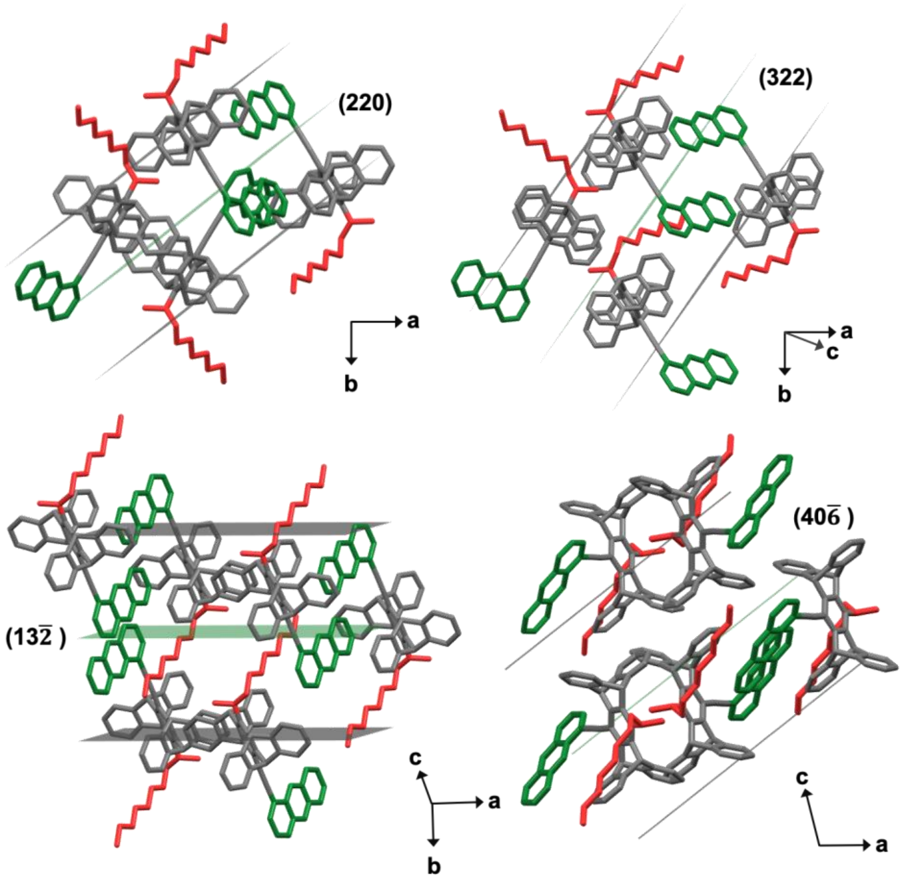

Figure S11. The (220), (322), (13$\bar{2}$), and (40$\bar{6}$) planes of **1G** corresponding to the PXRD signals at 2θ = 12.7°, 17.3°, 16.1°, and 19.1°, respectively.

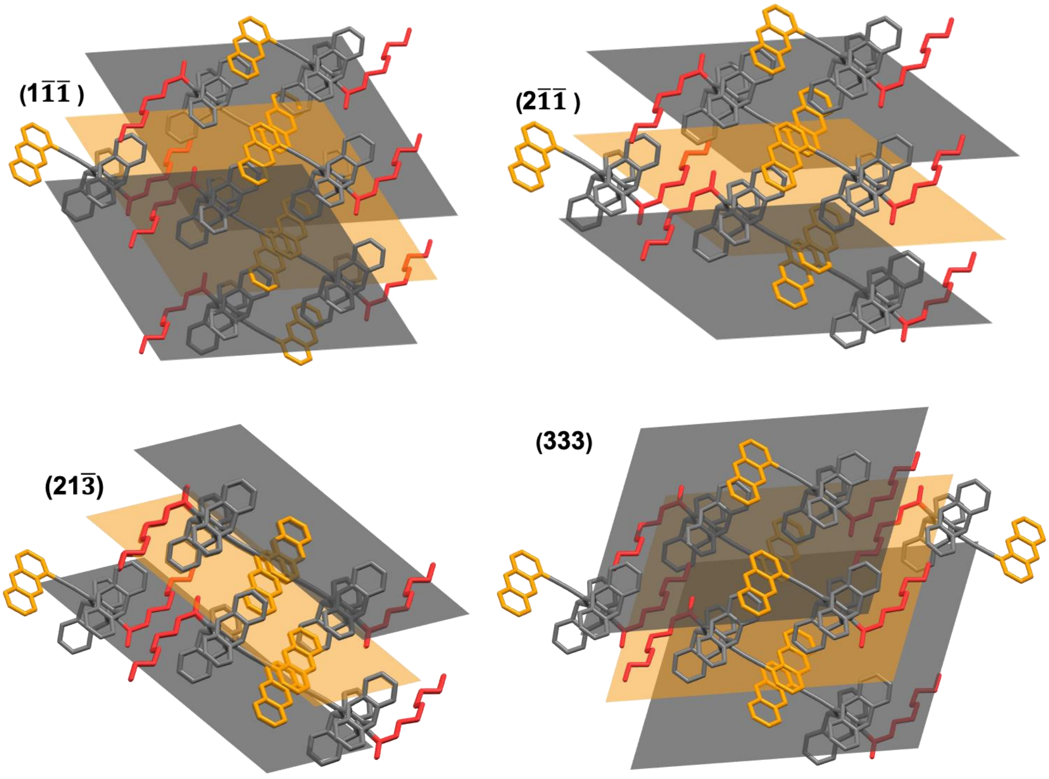

Figure S12. The (1$\bar{1}\bar{1}$), (2$\bar{1}\bar{1}$), (21$\bar{3}$), and (333) planes of **1Y** corresponding to the PXRD signals at 2θ = 8.9°, 14.6°, 17.7° and 24.3°, respectively.

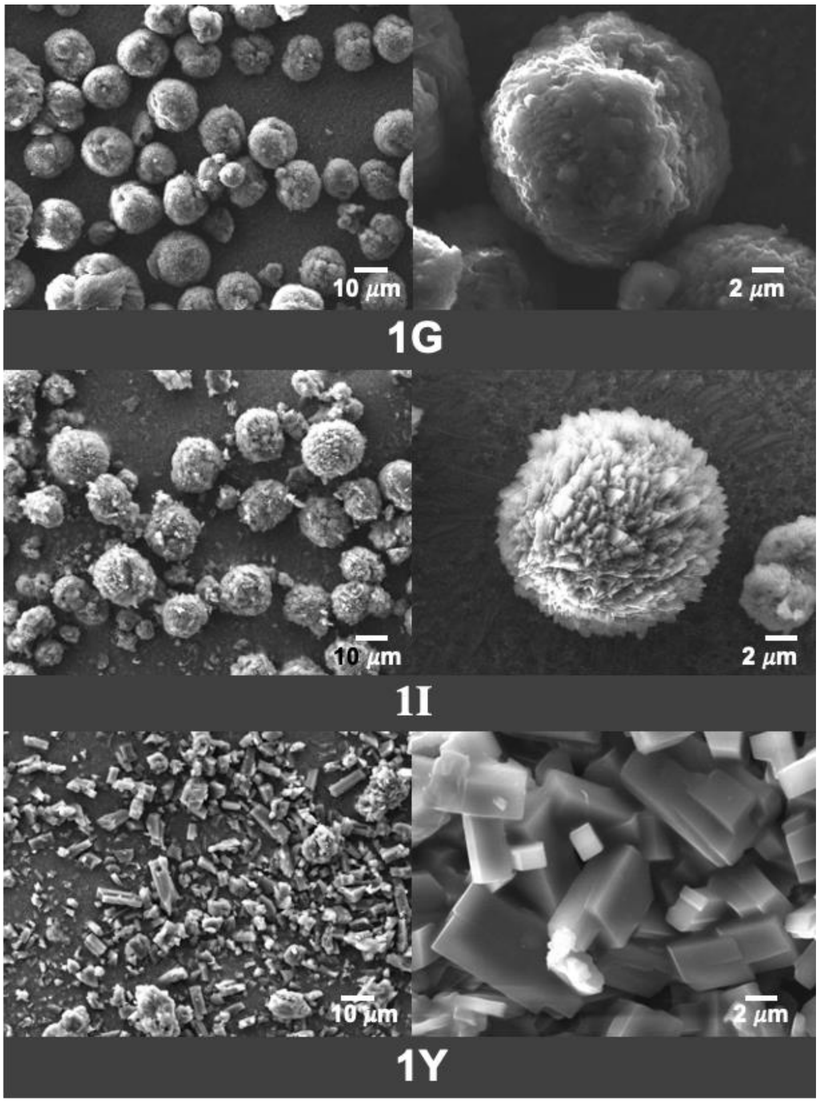

Figure S13. Scanning electron microscope (SEM) images of **1G**, **1I**, and **1Y (**from top to bottom) in polycrystalline powders.

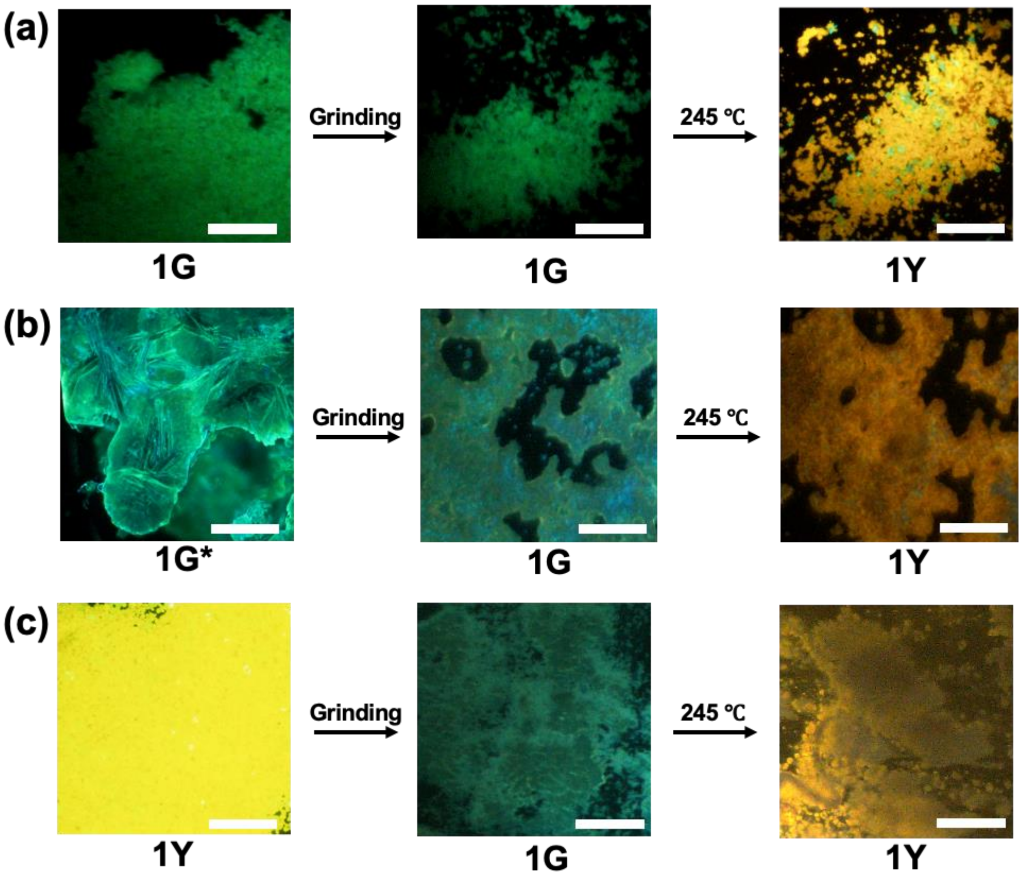

Figure S14. Fluorescence images (scale bars: 200 μm) of mechanical grinding followed by heating to 245°C for (a) **1G**, (b) **1G***, and (c) **1Y**.

NMR spectra

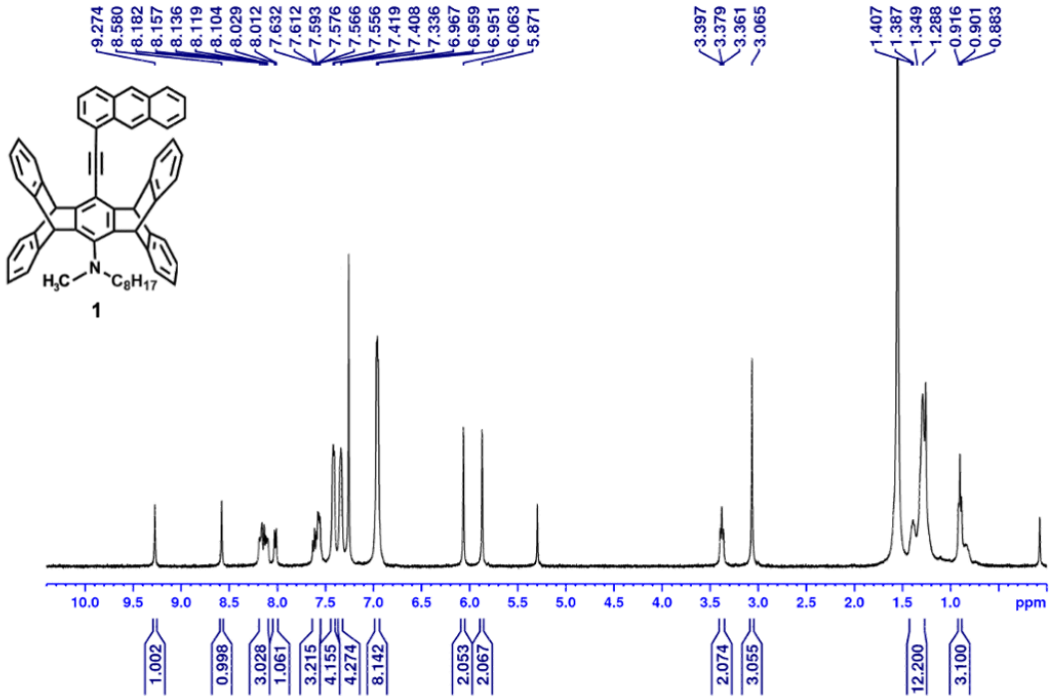

Figure S15. ^{1}H-NMR spectrum of compound **1** (400 MHz, $CDCl_3$).

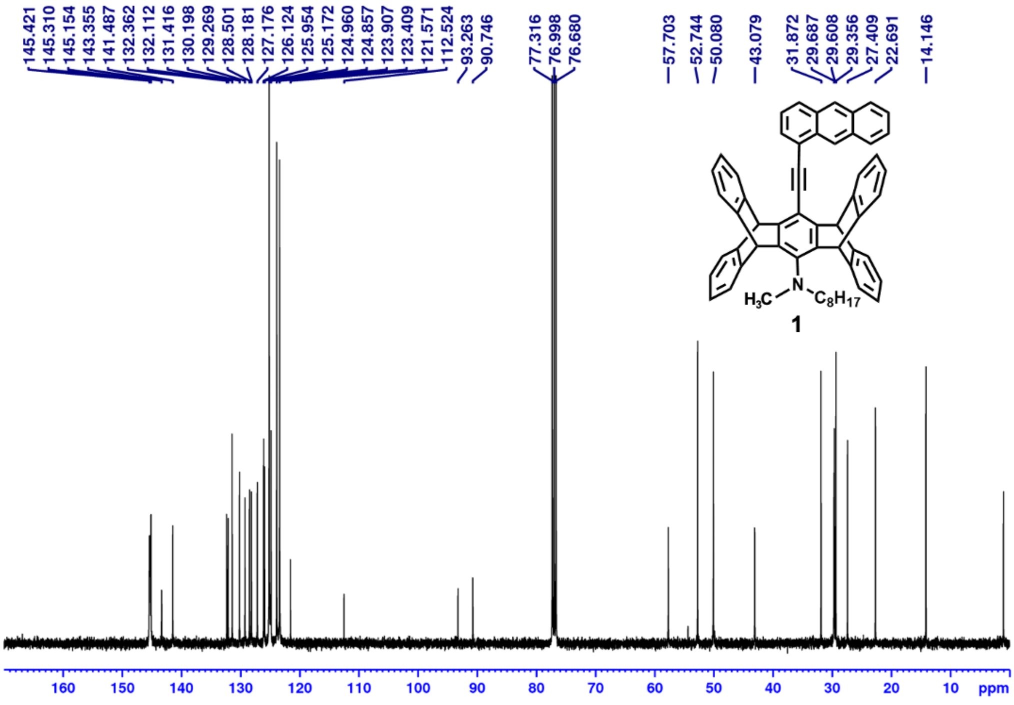

Figure S16. $^{13}C\{^{1}H\}$-NMR spectrum of compound **1** (100 MHz, $CDCl_3$).

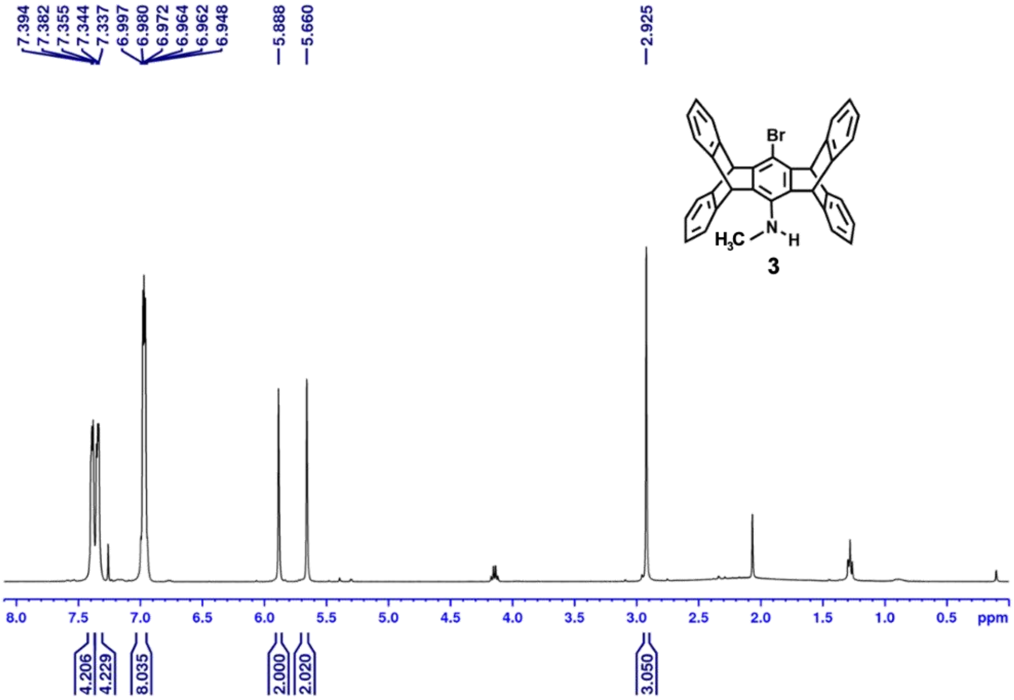

Figure S17. ^{1}H-NMR spectrum of compound **3** (400 MHz, $CDCl_3$).

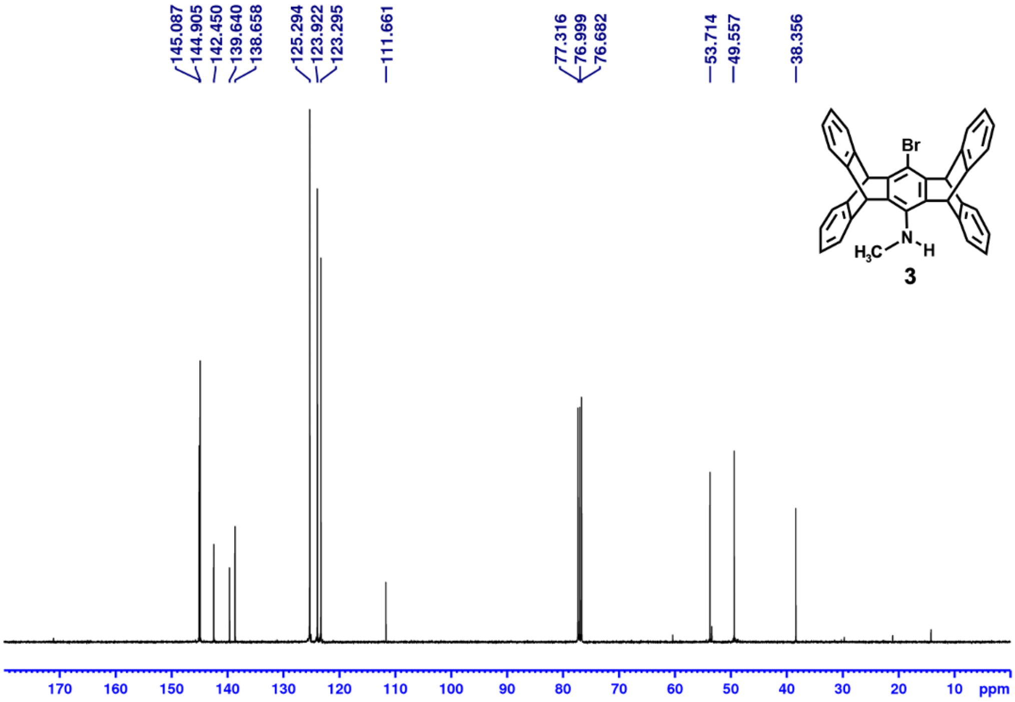

Figure S18. ^{13}C{^{1}H}-NMR spectrum of compound **3** (100 MHz, $CDCl_3$).

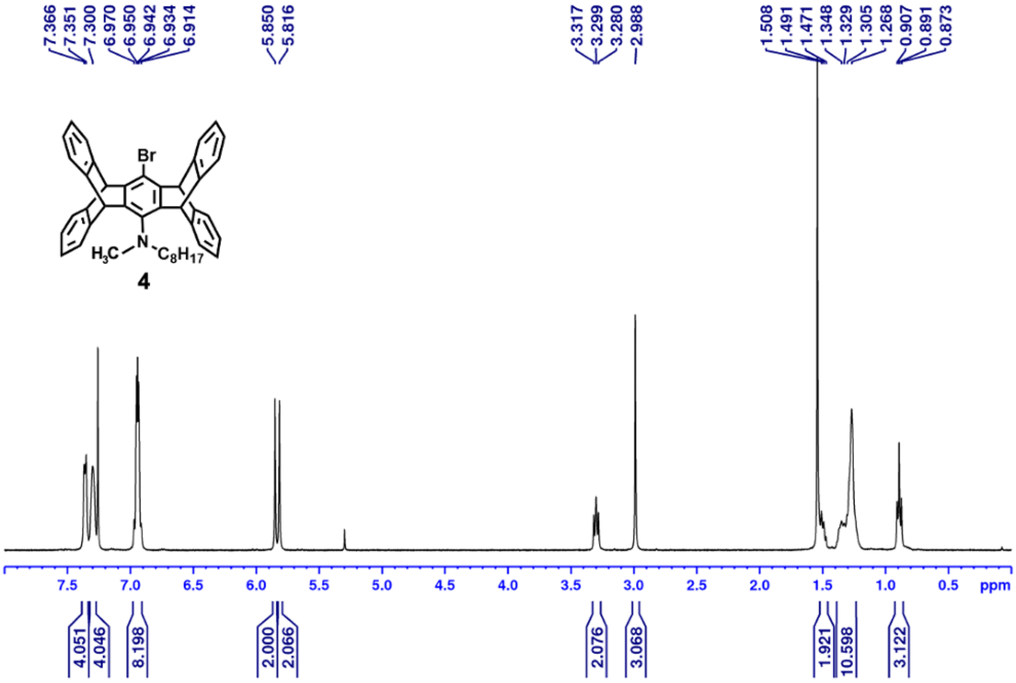

Figure S19. ^{1}H-NMR spectrum of compound **4** (400 MHz, $CDCl_3$).

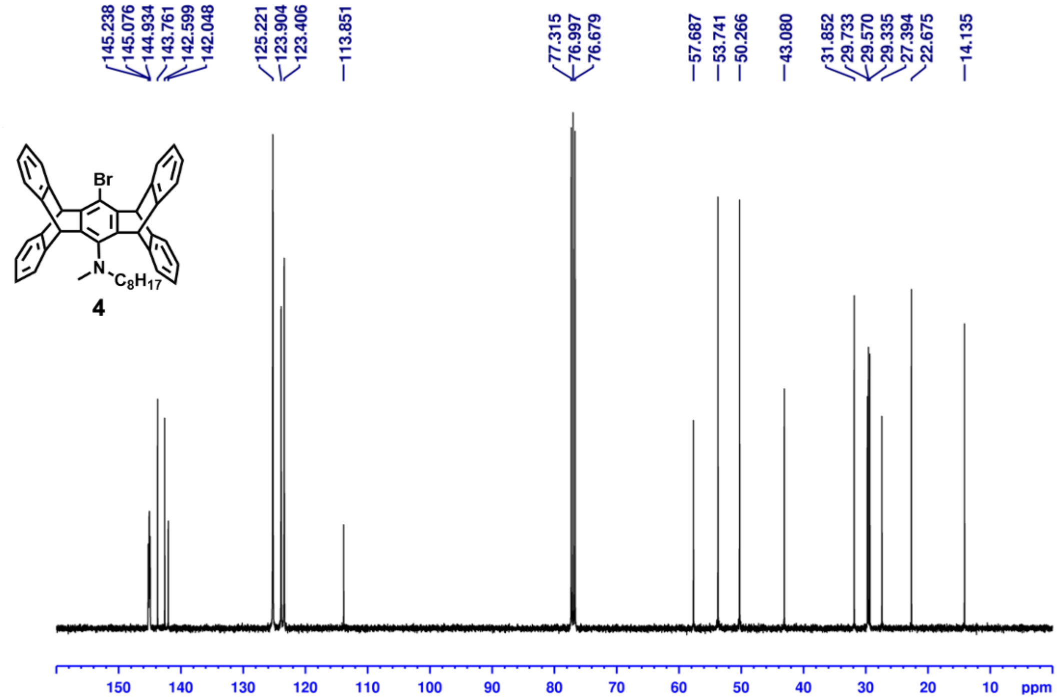

Figure S20. $^{13}C\{^{1}H\}$-NMR spectrum of compound **4** (100 MHz, $CDCl_3$).

Reference

(S1)Melhuish, W. The measurement of absolute quantum efficiencies of fluorescence. *New Zealand J. Sci. Technol* **1955**, *37*, 142.

(S2) Yang, J.-S.; Yan, J.-L.; Jin, Y.-X.; Sun, W.-T.; Yang, M.-C. Synthesis of New Halogenated Pentiptycene Building Blocks. *Org. Lett.* **2009**, *11* (6), 1429-1432.
